# BgeeDB, an R package for retrieval of curated expression datasets and for gene list expression localization enrichment tests

**DOI:** 10.12688/f1000research.9973.2

**Published:** 2018-08-07

**Authors:** Andrea Komljenovic, Julien Roux, Julien Wollbrett, Marc Robinson-Rechavi, Frederic B. Bastian

**Affiliations:** 1 Department of Ecology and Evolution, University of Lausanne, Lausanne, Switzerland; 2 SIB Swiss Institute of Bioinformatics, Lausanne, Switzerland; 3 Department of Biomedicine, University of Basel, Basel, Switzerland

**Keywords:** Bioconductor, R Package, Collective Data Access, Gene expression, Gene Enrichment Analysis

## Abstract

BgeeDB is a collection of functions to import into R re-annotated, quality-controlled and re-processed expression data available in the Bgee database. This includes data from thousands of wild-type healthy samples of multiple animal species, generated with different gene expression technologies (RNA-seq, Affymetrix microarrays, expressed sequence tags, and in situ hybridizations). BgeeDB facilitates downstream analyses, such as gene expression analyses with other Bioconductor packages. Moreover, BgeeDB includes a new gene set enrichment test for preferred localization of expression of genes in anatomical structures (“TopAnat”). Along with the classical Gene Ontology enrichment test, this test provides a complementary way to interpret gene lists.

Availability: https://www.bioconductor.org/packages/BgeeDB/

## Introduction

Gene expression levels influence the behavior of cells, the functionality of tissues, and a wide range of processes from development and aging to physiology or behavior. It is of particular importance that researchers are able to take advantage of the vast amounts of publicly available gene expression datasets to reproduce and validate results, or to investigate new research questions ^1– 3^.

To that purpose, one should be able to easily query and import gene expression datasets generated using different technologies, and their associated metadata. The R environment ^4^ has now become a standard for bioinformatics and statistical analysis of gene expression data, through the Bioconductor framework and its many open source packages ^5, 6^. It is thus desirable to provide an access to gene expression datasets programmatically and directly into R. For example, the Bioconductor packages ArrayExpress ^7^, GEOquery ^8^ and SRAdb ^9^ provide access to the reference databases ArrayExpress ^10^, GEO ^11^ and SRA ^12^ respectively.

However, such databases are primary archives aiming at comprehensiveness. They include gene expression datasets and other functional genomics data, generated from diverse experimental conditions, of diverse quality. The data provided are heterogeneous, with some datasets including only unprocessed raw data, and others including only data processed using specific analysis pipelines. For instance, over the 44,481 RNA array assay experiments stored in ArrayExpress with processed data available as of June 2018, 7,544 do not include the raw data. Metadata are often provided as free-text information that is difficult to query. For instance, the GEO database encourages submitters of high-throughput sequencing experiments to provide MINSEQE elements, but does not enforce this practice (see, e.g., GEO submission guidelines, and GEO Excel template for submissions). Unless the user needs to retrieve a specific known dataset from its accession number, it can be difficult to identify relevant available datasets. This can ultimately constitute an obstacle to data reuse.

One response to this diversity of primary archives is topical databases ^1^. They can be useful for researchers of specialized fields, and even more so if they propose an R package for data access. For example, the BrainStars Bioconductor package allows access to microarray data of mouse brain regions samples from the BrainStars project ^13, 14^. The ImmuneSpaceR Bioconductor package allows access to the gene expression data generated by the Human Immunology Project Consortium ^15^. Such efforts allow a better control of the data and annotation quality, but by nature they include a limited number of conditions, which only fit the needs of specialized projects. Similarly, numerous “ExperimentData” packages are available on the Bioconductor repository, which each include a single curated and well-formatted expression dataset (see https://www.bioconductor.org/packages/release/BiocViews.html#___ExpressionData). But these packages are rarely updated and are mostly meant to be used as examples in software packages vignettes, for teaching, or to provide supplementary data of publications. The package ExperimentHub ^16^ also provides access to a central location where this type of single datasets can be retrieved, but it does not address the difficulty of integrating datasets annotated and processed in different ways.

Finally, added-value databases aim at filtering, annotating, and possibly reprocessing all or some of the datasets available from the primary archives ^1^. For example, there is a Bioconductor package to access the Expression Atlas, which includes a selection of microarray and RNA-seq datasets from ArrayExpress that are re-annotated and reprocessed ^17, 18^. Similarly, the ReCount Bioconductor package provides access to a dataset of over 70,000 reanalyzed human RNA-seq samples from SRA (see https://jhubiostatistics.shinyapps.io/recount/) ^19– 21^.

The Bgee database ( https://bgee.org/) ^22^ is another added-value database, which currently offers access to reprocessed gene expression datasets from 29 animal species. Bgee aims at comparisons of gene expression patterns across tissues, developmental stages, ages and species. It provides manually curated annotations to ontology terms, describing precisely the experimental conditions used. It integrates expression data generated with multiple technologies: RNA-Seq, Affymetrix microarrays, *in situ* hybridization, and expressed sequence tags (ESTs) in release 14. An important characteristic of Bgee is that all datasets are manually curated to retain only “normal” healthy wild-type samples, i.e., excluding gene knock-out, treatments, or diseases. Finally, Bgee datasets are carefully checked for quality issues, and reprocessed to produce normalized expression level, calls of presence/absence of expression, and of differential expression. Bgee thus provides a reference of high-quality and reusable gene expression datasets that are relevant for biological insights into normal conditions of gene expression. The release 14.0 of Bgee covers 29 animal species, and includes 5,745 RNA-seq libraries, 12,996 Affymetrix chips, 360,653 results from 49,241 *in situ* hybridization experiments, and 3,335 EST libraries. This includes 4,860 human RNA-Seq libraries from the GTEx project ^23, 24^.

Until 2016 the Bgee database lacked a programmatic access to data through a R package, a shortcoming that we have addressed with the release of the BgeeDB Bioconductor package, available at https://www.bioconductor.org/packages/BgeeDB/. The package provides functions for fast extraction of data and metadata. The data structures used in the package can be easily incorporated with other Bioconductor packages, offering a wide range of possibilities for downstream analyses.

Moreover, we introduce in BgeeDB the possibility to run TopAnat analyses, i.e., anatomical expression enrichment tests on gene lists provided by the user. This functionality is based on the topGO package ^25, 26^, modified to use Bgee data (A. Alexa, personal communication). TopAnat is similar to the widely used Gene Ontology enrichment test ^27– 29^. But in our case, the enrichment test is applied to terms from an anatomical ontology, mapped to genes by expression patterns. The reference set of genes in a given species consists of all genes for which at least one "present" expression call is available in Bgee. The expression calls are propagated to parent anatomical structures by part_of and is_a relations, using the Uberon anatomical ontology ^30, 31^ (e.g., a gene expressed in the "hindbrain" is also considered expressed in the parent structure "brain"). Different algorithms, from TopGO, are available in TopAnat to account for the non-independence of anatomical structures, and avoid the over-representation of lowly-informative top-level terms. Enrichment of expression is tested for each anatomical structure independently with a Fisher exact test, and the resulting p-values for all anatomical structures are then corrected using a FDR correction ^32^. As a result, TopAnat allows to discover the tissues where a set of genes is preferentially expressed. This feature is available as a web-tool at https://bgee.org/?page=top_anat, but the R package offers more flexibility in the choice of input data and analysis parameters, and possibilities of inclusion within programs or pipelines.

There exist few other tools allowing to perform anatomical expression enrichment tests. For instance, the web-application Tissue Specific Expression Analysis (TSEA ^33^ based on methods from refs ^34, 35^) allows to perform such analyses, but only in human and mouse, while TopAnat can be used for any species integrated in Bgee (29 species as of Bgee release 14.0). For human, TSEA is based on the Genotype-Tissue Expression (GTEx) RNA-Seq dataset, while Bgee integrates GTEx data, but also other RNA-Seq datasets, and datasets from different data types, providing a higher diversity of anatomical structures. TSEA was last updated on March 2014. The database wormbase also proposes a similar tool, but for analyses only in C. elegans ^36^. There exists another application for expression enrichment analyses, but focused on analyzing gene regulatory networks in human ^37^.

The pipeline to process the data accessible through the BgeeDB package is documented in detail at https://github.com/BgeeDB/bgee_pipeline. In brief, for RNA-seq experiments: data present in SRA ^12^ are selected and annotated using information from GEO ^11^ or from papers, or provided by the Model Organism Database Wormbase ^38^. GTF annotation files and genome sequence fasta files are retrieved from Ensembl and Ensembl Genomes Metazoa ^39, 40^. After quality control steps, the Kallisto software is used to perform a pseudo-mapping of the reads to the transcriptome ^41^. TMM normalization ^42^ is used to normalize TPM and F/RPKM values within each experiment independently. Present/absent expression calls are produced for each library by comparing the level of expression of each gene to the background transcriptional noise in the library (estimated by using the level of expression of intergenic regions; Roux J., Rosikiewicz M., Wollbrett J., Robinson-Rechavi M., Bastian F.B.; in preparation). In brief, for Affymetrix experiments: data present in ArrayExpress and GEO are selected and annotated using the information available in these repositories, or in papers, or provided by the Model Organism Database Wormbase. Mappings of probesets to genes are retrieved from Ensembl and Ensembl Genomes Metazoa. Quality controls are performed to remove low quality chips and redundant chips ^43, 44^. When raw data are available, they are normalized using gcRMA (using version 2.42.0 for Bgee release 14.0) within each experiment independently ^45^. Present/absent expression calls are generated either from the MAS5 processed data ^46^, based on the perfect match/mismatch probesets, or using the raw data when available, by comparing the signal of a probeset to a subset of weakly expressed probesets ^47^.

The BgeeDB package information is available on the Bioconductor website at https://bioconductor.org/packages/BgeeDB/. The source code is available at https://github.com/BgeeDB/BgeeDB_R. The preferred location for filing bug reports and suggestions is the issue tracker on GitHub.

In the following sections we provide some typical examples of usage of the BgeeDB package.

## Methods

### Requirements

To reproduce the results of examples in this paper, based on Bgee release 14.0:

- R >= 3.5
- Bioconductor >= 3.7
- BgeeDB package version >= 2.6.2
- edgeR = 3.22.2
- Mfuzz = 2.40.0
- biomaRt >= 2.36.1 (with Ensembl release 84 accessible)
- Working internet connection

Please note that an earlier version of Bioconductor and of R (>= 3.3) could be used, but would require to clone our GitHub repository and to use the R CMD BUILD command to build the package.

### Package installation



source("https://bioconductor.org/biocLite.R")
biocLite("BgeeDB")
# load the library
library(BgeeDB)
                    


## Use cases

### Data download and import of normalized expression levels

The first step of data retrieval is to initialize a new Bgee reference class object, for a targeted species and data type. Normalized expression levels are currently available in the BgeeDB package for two data types: Affymetrix microarrays and Illumina RNA-seq. The list of species available in the Bgee database for each data type, along with their NCBI taxonomy IDs and common names can be obtained with the listBgeeSpecies() function. By default, data will be downloaded from the latest Bgee release, but this can be changed with the release argument.

Next, the functions getAnnotation(), getData(), and formatData() can be called to respectively download the annotations of datasets, download the actual expression data, and reformat the expression data for more convenient use. Of note, BgeeDB creates a directory to store the downloaded annotation files and datasets, by default in the user’s R working directory, but this can be changed with the pathToData argument. These versioned cached files make it faster for the user to return to previously used data and allow to work offline.

***Microarray dataset retrieval.*** In the following example, we will look for a microarray dataset in mouse ( *Mus musculus*), spanning multiple early developmental stages, including zygote.



# specify species and data type
# the examples in this paper are based on Bgee release 14.0
# the following line targets the latest Bgee release.
# In order to target specifically the release 14.0,
# add the parameter 'release="14.0"'

bgee.affymetrix <- Bgee$new(species="Mus_musculus", dataType="affymetrix")

# retrieve annotation of all mouse affymetrix datasets in Bgee
annotation.bgee.mouse.affymetrix <- getAnnotation(bgee.affymetrix)

                    


This creates a list of two data frames, one including the annotation of experiments, and the other including the annotation of each individual sample, i.e., hybridized microarray chip. For mouse, there are 698 Affymetrix experiments and 6,095 samples available in Bgee release 14.0. Anatomical structures and developmental stages are annotated using the Uberon ontology ^30, 31^. Sex and strain information is also provided. Below, we are selecting the experiments for which at least one sample is annotated to the zygote stage ( UBERON:0000106).



# retrieve annotations of samples and experiments
sample.annotation <- annotation.bgee.mouse.affymetrix$sample.annotation
experiment.annotation <- annotation.bgee.mouse.affymetrix$experiment.annotation

# list experiments including a zygote sample 
selected.experiments <- unique(sample.annotation$Experiment.ID[sample.annotation$Stage.ID == "UBERON:0000106"])
experiment.annotation[experiment.annotation$Experiment.ID %in% selected.experiments,]

# stages sampled in each of these experiments
unique(sample.annotation[sample.annotation$Experiment.ID %in% selected.experiments, c("Experiment.ID", "Stage.name")])
                    


This yields three microarray experiments, with accessions GSE1749, E-MEXP-51 and GSE18290. Among these, the accession E-MEXP-51, submitted to ArrayExpress by Wang and colleagues ^48^, includes samples from more developmental stages than the other two, so we will choose this one for the next steps. For this experiment, raw data were available from ArrayExpress, so samples were fully normalized with gcRMA ^49^ trough the Bgee pipeline.



# List all samples from E-MEXP-51 in Bgee
sample.annotation[sample.annotation$Experiment.ID == "E-MEXP-51",]
                    


The experiment includes 35 samples that passed Bgee quality controls. They originate from 12 developmental stages: primary and secondary oocyte, zygote, early, mid and late 2-cells embryo, 4-cells embryo, 8-cells embryo, 16-cells embryo, early, mid and late blastocyst. The developmental stage ontology used is not precise enough yet to differentiate some of these conditions: the early, mid and late 2-cells stages are annotated as Theiler stage 2 embryo, and the 4-cells and 8-cells stages are annotated as Theiler stage 3 embryo. All samples were hybridized to the Affymetrix GeneChip Murine Genome U74Av2 microarray. Let us download the normalized probesets intensities measured for all samples.



data.E.MEXP.51 <- getData(bgee.affymetrix, experimentId="E-MEXP-51")
head(data.E.MEXP.51)
                    


The resulting data frame lists for each sample (column “Chip.ID”), the 8,954 probesets on the microarray (column “Probeset.ID”), their mapping to Ensembl gene IDs (column “Gene.ID”), their logged normalized intensities (column “Log.of.normalized.signal.intensity”), and a presence/absence call and quality (columns “Detection.flag” and “Detection.quality”).

As this format might not be the most convenient for downstream processing of an expression dataset, we offer the formatData() function, which creates an ExpressionSet object including the expression data matrix, the probesets annotation to Ensembl genes and the samples' anatomical structure and stage annotation into ( assayData, featureData and phenoData slots respectively). This object class is of standard use in numerous Bioconductor packages.



data.E.MEXP.51.formatted <- formatData(bgee.affymetrix, data.E.MEXP.51,
callType="all", stats="intensities")
data.E.MEXP.51.formatted
# matrix of expression intensities
head(exprs(data.E.MEXP.51.formatted))
# annotation of samples
pData(data.E.MEXP.51.formatted)
# annotation of probesets
head(fData(data.E.MEXP.51.formatted))
                    


The callType option of the formatData() function could alternatively be set to present or present high quality to display only the intensities of probesets detected as actively expressed.

The result is a nicely formatted Bioconductor object including expression data and their annotations, ready to be used for downstream analysis with other Bioconductor packages.

***RNA-seq dataset retrieval.*** We will now search Bgee for a RNA-seq dataset sampling brain and liver tissues (Uberon Ids UBERON:0000955 and UBERON:0002107 respectively) in macaque ( *Macaca mulatta*), and including multiple biological replicates for each tissue. As for Affymetrix data, Bgee RNA-seq annotations provide information about anatomical structure, developmental stage, sex, and strain.



# specify species and data type
# the examples in this paper are based on Bgee release 14.0
# the following line targets the latest Bgee release. In order
# to target specifically the release 14.0, add the parameter
# 'release="14.0"'
bgee.rnaseq <- Bgee$new(species="Macaca_mulatta", dataType="rna_seq")

# retrieve annotations of RNA-seq samples and experiments
annotation.bgee.macaque.rna.seq <- getAnnotation(bgee.rnaseq)
sample.annotation <- annotation.bgee.macaque.rna.seq$sample.annotation
experiment.annotation <- annotation.bgee.macaque.rna.seq$experiment.annotation

# list experiments including both brain and liver samples
selected.experiments <- intersect(unique(sample.annotation$Experiment.ID[sample.annotation$Anatomical.entity.ID == "UBERON:0000955"]),
unique(sample.annotation$Experiment.ID[sample.annotation$Anatomical.entity.ID == "UBERON:0002107"]))
experiment.annotation[experiment.annotation$Experiment.ID %in% selected.experiments,]

# check whether experiments include biological replicates
sample.annotation[sample.annotation$Experiment.ID %in%
selected.experiments & (sample.annotation$Anatomical.entity.ID == "UBERON:0000955" 
| sample.annotation$Anatomical.entity.ID == "UBERON:0002107"), c("Experiment.ID","Library.ID","Anatomical.entity.ID", "Anatomical.entity.name","Stage.ID")]
                    


Accessions GSE41637 ^50^ and GSE30352 ^51^ both include biological replicates for brain and liver. We will focus on GSE41637 for the next steps since it includes three replicates of each tissue, vs. only two for GSE30352. We will download the dataset and reformat it to obtain an ExpressionSet including counts of mapped reads on each Ensembl gene for each sample.



data.GSE41637 <- getData(bgee.rnaseq, experimentId="GSE41637")
data.GSE41637.formatted <- formatData(bgee.rnaseq, data.GSE41637, callType="all", stats="counts")
data.GSE41637.formatted
                    


Instead of mapped read counts, it is also possible to fill the data matrix with expression levels in F/RPKMs (fragments/reads per kilobase per million reads) or in TPM (transcript per million) ^52, 53^, using the option stats="fpkm" or stats="tpm".

***Presence/absence calls retrieval.*** It is often difficult to compare expression levels across species ^54^, and even within species, across datasets generated by different experimenters or laboratories ^55– 57^. Batch effects have indeed been shown to impact extensively gene expression levels, confounding biological signal differences.

Encoding gene expression as present or absent in a sample allows a more robust comparison across such conditions. In addition to retrieving RNA-seq and Affymetrix quantitative expression levels, BgeeDB also allows to retrieve calls of presence or absence of expression computed in the Bgee database for each gene (RNA-seq) or probeset (Affymetrix), in the column “Detection.flag” of the data.E.MEXP.51 and data.GSE41637 objects created above. And interestingly, expression calls are also available in Bgee for ESTs and *in situ* hybridization data, as well as for the consensus of the four data types for each combination “gene / tissue / developmental stage / sex / strain”.

A powerful use of these expression calls is the anatomical expression enrichment test “TopAnat”. TopAnat uses a similar approach to Gene Ontology enrichment tests ^27^, but genes are associated to the anatomical structures where they display expression, instead of to their functional classification. These tests allow discovering where a set of genes is preferentially expressed as compared to a background universe (Roux J., Seppey M., Sanjeev K., Rech de Laval V., Moret P., Artimo P., Duvaud S., Ioannidis V., Stockinger H., Robinson-Rechavi M., Bastian F.B.; in preparation). We show an example of such an analysis in the section “Anatomical expression enrichment analysis” below.

Of note, the expression calls imported from BgeeDB can also be used for other downstream analyses. For example, when studying protein-protein interaction datasets, it might be biologically relevant to retain only interactions for which both members are expressed in the same tissues ^58, 59^.

### Downstream analysis examples

***Clustering analysis.*** A variety of downstream analyses can be performed on the imported expression data. Below we detail an example of gene expression clustering analysis on the developmental time-series microarray experiment imported above. The analysis, performed with the Mfuzz package ^60, 61^ (version 2.40.0 for this paper), aims at uncovering genes with similar expression profiles across development. We can readily start with the ExpressionSet object previously created.



# for simplicity, keep only one sample per condition
data.E.MEXP.51.formatted <- data.E.MEXP.51.formatted[,!duplicated(pData(data.E.MEXP.51.formatted)[
c("Anatomical.entity.ID","Anatomical.entity.name","Stage.ID","Stage.name")])]

# order developmental stages
stages <- c("GVoocyte1","MIIoocyte1","Zygote1","Early2-cell1","4Cell1","16cell1","EarlyBlastocyst1","MidBlastocyst1",
"LateBlastocyst1")
data.E.MEXP.51.formatted <- data.E.MEXP.51.formatted[, stages]

# filter out rows with no variance
data.E.MEXP.51.formatted <-
data.E.MEXP.51.formatted[apply(exprs(data.E.MEXP.51.formatted), 1, sd) != 0, ]

# Mfuzz clustering
biocLite("Mfuzz")
library(Mfuzz)
# standardize matric of expression data
z.mat <- standardise(data.E.MEXP.51.formatted)
# cluster data into 16 clusters
clusters <- mfuzz(z.mat, centers=16, m=1.25)

# visualizing clusters
mfuzz.plot2(z.mat, cl=clusters, mfrow=c(4,4), colo="fancy",
time.labels=row.names(pData(z.mat)), las=2, xlab="", ylab="Standardized expression level", x11=FALSE)
                    


**Figure 1.**
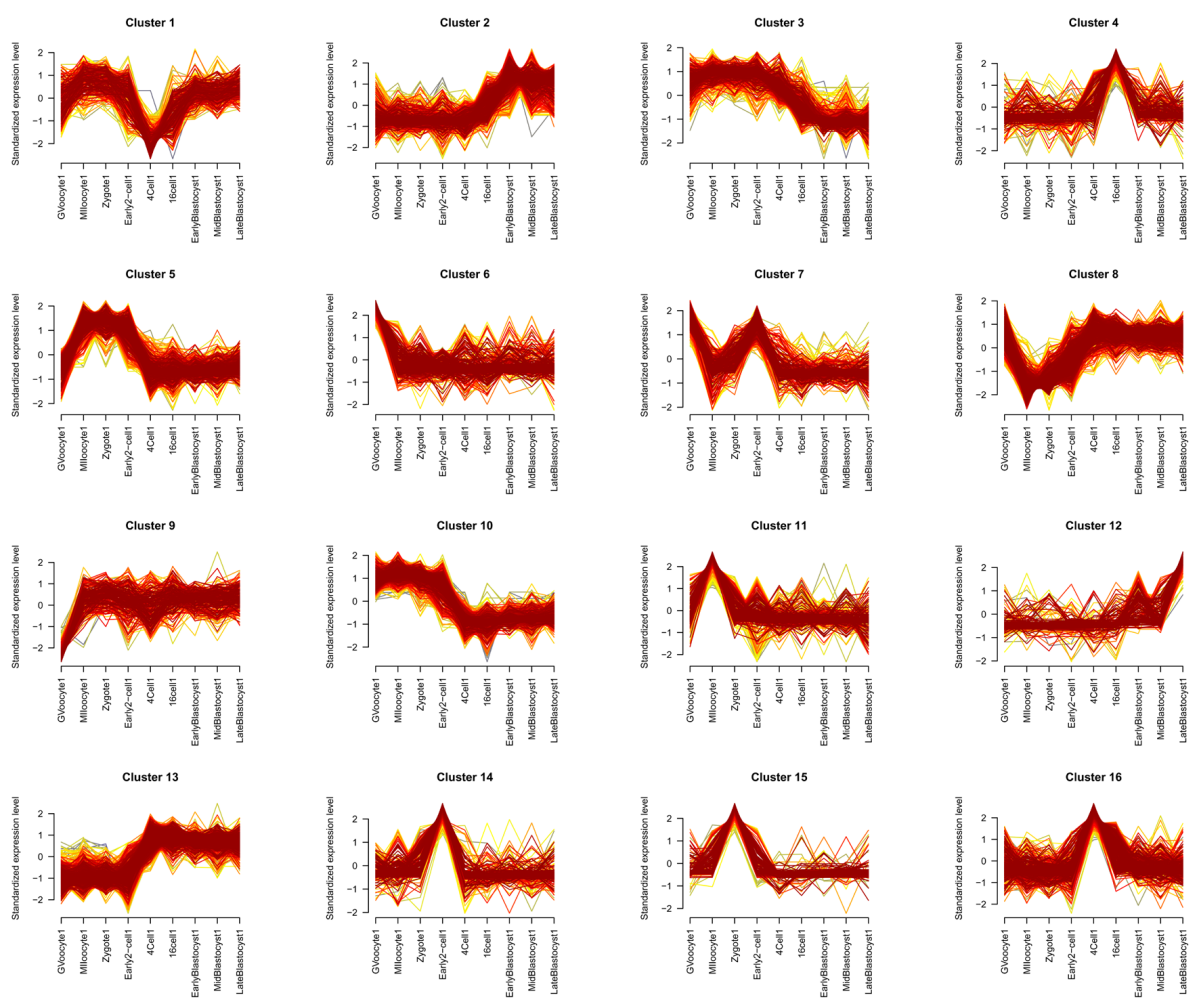
Standardized expression levels of 16 groups of microarray probesets, clustered according to their expression during mouse early development. The x-axis displays sample names (column “Chip.ID” of the data.E.MEXP.51 object).

The resulting plot can be seen in Figure 1.

***Differential expression analysis.*** Below, we detail a differential expression analysis, with the package edgeR ^62, 63^ (version 3.22.2 for this paper), on the previously imported RNA-seq dataset of macaque tissues. We aim at isolating genes differentially expressed between brain and liver.



# differential expression analysis with edgeR
biocLite("edgeR")
library(edgeR)

# subset the dataset to brain and liver
brain.liver <- data.GSE41637.formatted[, pData(data.GSE41637.formatted)$Anatomical.entity.name %in%
c("brain", "liver")]  

# filter out very lowly expressed genes
brain.liver.filtered <- brain.liver[rowSums(cpm(brain.liver) > 1) > 3, ]

# create edgeR DGElist object
dge <- DGEList(counts=brain.liver.filtered,
group=pData(brain.liver.filtered)$Anatomical.entity.name)
dge <- calcNormFactors(dge)
dge <- estimateCommonDisp(dge)
dge <- estimateTagwiseDisp(dge)
de <- exactTest(dge, pair=c("brain","liver"))
de.genes <- topTags(de, n=nrow(de))$table

# MA plot with DE genes highlighted
plotSmear(dge, de.tags=rownames(de.genes)[de.genes$FDR < 0.01], cex=0.3)
                    


**Figure 2.**
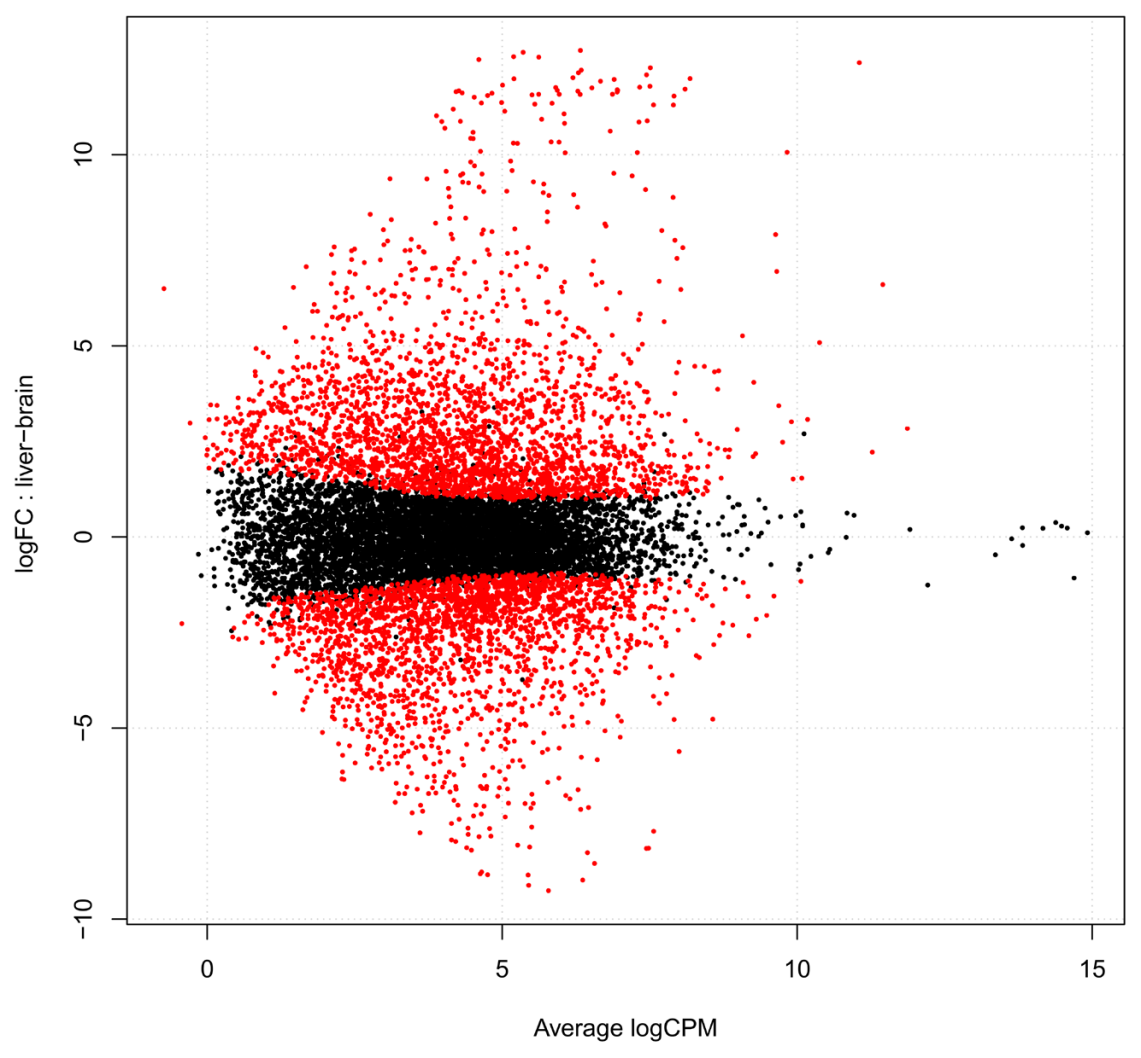
Mean-average (MA) plot of differential gene expression between brain and liver in macaque based on RNA-seq data. Significantly differentially expressed genes (FDR < 1%) are highlighted in red.

The resulting plot can be seen in Figure 2.

### Anatomical expression enrichment analysis

The loadTopAnatData() function loads the names of anatomical structures, and relationships between them, from the Uberon anatomical ontology (based on parent-child “is_a” and “part_of” relationships). It also loads a mapping from genes to anatomical structures, based on the presence calls of the genes in the targeted species. These calls come from a consensus of all data types specified in the input Bgee class object. We recommend to use all available data types (in Bgee 14, RNA-seq, Affymetrix, EST and *in situ* hybridization) for both genomic coverage and anatomical precision, which is the default behavior if no dataType argument is specified when the Bgee class object is created.

By default, presence calls of both silver and gold quality are used, which can be changed with the confidence argument of the loadTopAnatData() function (in releases of Bgee up to 13, "high" and "low" confidence were used). Finally, it is possible to specify the developmental stage under consideration, with the stage argument. By default expression calls generated from samples of all developmental stages are used, which is equivalent to specifying stage="UBERON:0000104" (“life cycle”, the root of the stage ontology). Data are stored in versioned tab-separated cached files that will be read again if a query with the exact same parameters is launched later, to save time and server resources, and to work offline.

In this example, we will use expression calls for zebrafish genes using all sources of expression data.



# the examples in this paper are based on Bgee
# release 14.0
# the following line targets the latest Bgee release. In order to target
# specifically the release 14.0, add the parameter 'release="14.0"'
bgee.topanat <- Bgee$new(species="Danio_rerio")
top.anat.data <- loadTopAnatData(bgee.topanat)
                    


We will look at the expression localization of the genes with an annotated phenotype related to pectoral fin (i.e., genes which upon knock-out or knock-down led to abnormal phenotypes of pectoral fin or its components). Zebrafish phenotypic data are available from the ZFIN database ^64^ and integrated into the Ensembl database ^39^. We will thus retrieve the targeted genes using the biomaRt ^65^ Bioconductor package (version 2.36.1 for this paper).



biocLite("biomaRt")
library(biomaRt)

# zebrafish data in Ensembl 84, that Bgee 14.0 uses (stable link)
ensembl <- useMart("ENSEMBL_MART_ENSEMBL",
dataset="drerio_gene_ensembl", host="mar2016.archive.ensembl.org")

# get the mapping of Ensembl genes to phenotypes
genes.to.phenotypes <- getBM(filters=c("phenotype_source"), value=c("ZFIN"),
attributes=c("ensembl_gene_id","phenotype_description"), mart=ensembl)

# select phenotypes related to pectoral fin
Phenotypes <- grep("pectoral fin", unique(genes.to.phenotypes$phenotype_description), value=T)

# select the genes annotated to select phenotypes
genes <- unique(genes.to.phenotypes$ensembl_gene_id[
genes.to.phenotypes$phenotype_description %in% phenotypes])
                    


This gives a list of 147 zebrafish genes implicated in the development and function of pectoral fin. The next step of the analysis relies on the topGO Bioconductor package. We will prepare a modified topGOdata object allowing to handle the Uberon anatomical ontology instead of the Gene Ontology, and perform a GO-like enrichment test for anatomical terms. As for a classical topGO analysis, we need to prepare a vector including all background genes, and with values 0 or 1 depending if genes are part of the foreground or not. The choice of background is very important since the wrong background can lead to spurious results in enrichment tests ^66^. Here we choose as background all zebrafish Ensembl genes with an annotated phenotype from ZFIN.



# prepare the gene list vector 
gene.list  <- factor(as.integer(unique(genes.to.phenotypes$ensembl_gene_id) %in% genes))
names(gene.list) <- unique(genes.to.phenotypes$ensembl_gene_id)
summary(gene.list)

# prepare the topAnat object based on topGO
top.anat.object  <- topAnat(top.anat.data, gene.list)
top.anat.object
                    


At this step, expression calls are propagated through the whole ontology (e.g., expression in the forebrain will also be counted as expression in the brain, the nervous system, etc). This can take some time, especially if the gene list is large.

Finally, we can launch an enrichment test for anatomical terms. The functions of the topGO package can directly be used at this step. See the vignette of this package for more details ^26^. Here we will use a Fisher test, coupled with the “weight” decorrelation algorithm.



results <- runTest(top.anat.object, algorithm='weight', statistic='fisher')
results
                    


Finally, we implemented a function to display results in a formatted table. By default anatomical structures are sorted by their test *p*-value, which is displayed along with the associated false discovery rate (FDR ^32^) and the enrichment fold. Sorting on other columns of the table (e.g., on decreasing enrichment folds) is possible with the ordering argument. Of note, it is debated whether a FDR correction is relevant on such enrichment test results, since tests on different terms of the ontologies are not independent. An interesting discussion can be found in the vignette of the topGO package.



# retrieve anatomical structures enriched at a 1% FDR threshold
table.Over <- makeTable(top.anat.data, top.anat.object, results, cutoff=0.01)
head(table.over)
                    


The 27 anatomical structures displaying a significant enrichment at a FDR threshold of 1% are show in Table 1. The first term is “pectoral fin”, and the second “paired limb/fin bud”. Other terms in the list, especially those with high enrichment folds, are clearly related to pectoral fins (e.g., “pectoral appendage field”), or substructures of fins (e.g., “fin bone”). This analysis shows that genes with phenotypic effects on pectoral fins are specifically expressed in or next to these structures. More generally, it demonstrates the relevance of TopAnat analysis for the characterization of lists of genes.

Of note, it is possible to retrieve for a particular tissue the significant genes that were mapped to it.



# In order to retrieve significant genes mapped to the term "paired limb/fin bud"
term <- "UBERON:0004357"
termStat(top.anat.object, term) 

# 198 genes mapped to this term for Bgee 14.0 and Ensembl 84
genesInTerm(top.anat.object, term)
# 48 significant genes mapped to this term for Bgee 14.0
# and Ensembl 84
annotated <- genesInTerm(top.anat.object,
term)[["UBERON:0004357"]]
annotated[annotated %in% sigGenes(top.anat.object)]
                    


**Table 1.** Zebrafish anatomical structures showing a significant enrichment in expression of genes with a pectoral fin phenotype (FDR < 1%). The “weight” algorithm of the topGO package was used to decorrelate the structure of the ontology.

organId	organName	annotated	significant	expected	foldEnrichment	pValue	FDR
UBERON:0000151	pectoral fin	439	79	21.48	3.68	1.36E-27	1.47E-24
UBERON:0004357	paired limb/fin bud	198	48	9.69	4.95	5.19E-23	2.80E-20
UBERON:2000040	median fin fold	59	20	2.89	6.92	9.37E-13	3.38E-10
UBERON:0003051	ear vesicle	391	49	19.13	2.56	5.50E-11	1.49E-08
UBERON:0005729	pectoral appendage field	20	11	0.98	11.22	3.05E-10	6.60E-08
UBERON:0004376	fin bone	34	12	1.66	7.23	2.60E-08	4.69E-06
UBERON:0011004	pharyngeal arch cartilage	66	16	3.23	4.95	4.96E-08	7.65E-06
UBERON:0003406	cartilage of respiratory system	52	14	2.54	5.51	8.61E-08	1.16E-05
UBERON:0004756	dermal skeletal element	55	15	2.69	5.58	9.14E-07	1.10E-04
UBERON:0003108	suspensorium	56	13	2.74	4.74	1.66E-06	1.79E-04
UBERON:0004375	bone of free limb or fin	27	9	1.32	6.82	2.77E-06	2.72E-04
UBERON:0001042	chordate pharynx	417	44	20.40	2.16	3.38E-06	3.04E-04
UBERON:0006068	bone of tail	11	6	0.54	11.11	4.67E-06	3.89E-04
UBERON:0003128	cranium	334	37	16.34	2.26	5.25E-06	4.05E-04
UBERON:4000170	median fin skeleton	26	8	1.27	6.30	2.00E-05	1.44E-03
UBERON:0004117	pharyngeal pouch	51	11	2.49	4.42	2.32E-05	1.57E-03
UBERON:0002533	post-anal tail bud	1447	95	70.79	1.34	2.77E-05	1.76E-03
UBERON:0012275	meso-epithelium	1616	104	79.05	1.32	5.53E-05	3.32E-03
UBERON:0002514	intramembranous bone	23	7	1.13	6.19	7.36E-05	4.01E-03
UBERON:0001708	jaw skeleton	108	24	5.28	4.55	7.77E-05	4.01E-03
UBERON:0001003	skin epidermis	112	16	5.48	2.92	7.79E-05	4.01E-03
UBERON:0002541	germ ring	117	16	5.72	2.80	1.33E-04	6.54E-03
UBERON:0010188	protuberance	598	68	29.25	2.32	1.51E-04	7.01E-03
UBERON:4000163	anal fin	12	5	0.59	8.47	1.57E-04	7.01E-03
UBERON:0010363	endochondral element	58	13	2.84	4.58	1.62E-04	7.01E-03
UBERON:2000555	opercular flap	26	7	1.27	5.51	1.74E-04	7.25E-03
UBERON:0007812	post-anal tail	1452	96	71.03	1.35	2.03E-04	8.13E-03

## Conclusion

In summary, the BgeeDB package serves as a bridge between curated data from the Bgee database and the R/Bioconductor environment, facilitating the access to high-quality curated and re-analyzed gene expression datasets, and significantly reducing time for downstream analyses of the datasets. Moreover, it provides access to TopAnat, a new enrichment tool allowing to make sense of lists of genes, by uncovering their preferential localization of expression in anatomical structures. The TopAnat workflow is straightforward; for users already using topGO in their analysis pipelines, performing a TopAnat analysis on the same gene list only requires 6 additional lines of code.

## Software and data availability

Software available from: http://www.bioconductor.org/packages/BgeeDB/

Latest source code: https://github.com/BgeeDB/BgeeDB_R

Archived source code as at the time of publication: https://doi.org/10.5281/zenodo.1293418 ^67^

## Supplementary material

File S1. R markdown file including code from the paper.

.

File S2. PDF file including the results of execution of the code from File S1.
