# Supplementary material for: BgeeDB, an R package for retrieval of curated expression datasets and for gene list expression localization enrichment tests
Source: F1000Res. 2018 Aug 7;5:2748. Originally published 2016 Nov 23. [Version 2] doi: 10.12688/f1000research.9973.2 (PMC6113886; doi:10.12688/f1000research.9973.2)
Supplement: Supplementary file 2 [file f1000research-5-16883-s0001.tgz › 734673b9-f41e-4294-b339-62acfabdd616.pdf]

Supplementary code (update) for article: BgeeDB, an R package for retrieval of curated expression datasets and for gene list enrichment tests

Andrea Komljenović, Julien Roux, Julien Wollbrett, Marc Robinson-Rechavi and Frederic Bastian

June 11, 2018

```
source("https://bioconductor.org/biocLite.R")
biocLite("BgeeDB")
```

```
##
## The downloaded binary packages are in
##  /var/folders/k2/897tjbxn5xd2gd228p8s5s3r0000gp/T//RtmpkIsKKI/downloaded_pac
kages
```

```
# additional packages for installation
biocLite(c("edgeR", "Mfuzz", "biomaRt"))
```

```
##
## The downloaded binary packages are in
##  /var/folders/k2/897tjbxn5xd2gd228p8s5s3r0000gp/T//RtmpkIsKKI/downloaded_pac
kages
```

```
library(BgeeDB)
```

```
# list the species
listBgeeSpecies()
```

```
##
## Querying Bgee to get release information...
##
## Building URL to query species in Bgee release 14...
##
## Submitting URL to Bgee webservice... (https://r.bgee.org/?page=r_package&act
ion=get_all_species&display_type=tsv&source=BgeeDB_R_package&source_version=2.6
.2)
##
## Query to Bgee webservice successful!
```

```
##        ID             GENUS     SPECIES_NAME        COMMON_NAME AFFYMETRIX
## 1   6239  Caenorhabditis          elegans           nematode       TRUE
## 2   7217      Drosophila        ananassae                         FALSE
## 3   7227      Drosophila     melanogaster          fruit fly       TRUE
## 4   7230      Drosophila       mojavensis                         FALSE
## 5   7237      Drosophila    pseudoobscura                         FALSE
## 6   7240      Drosophila         simulans                         FALSE
## 7   7244      Drosophila          virilis                         FALSE
## 8   7245      Drosophila           yakuba                         FALSE
## 9   7955           Danio            rerio          zebrafish       TRUE
## 10  8364         Xenopus       tropicalis western clawed frog      FALSE
## 11  9031          Gallus           gallus            chicken      FALSE
## 12  9258 Ornithorhynchus         anatinus           platypus      FALSE
## 13  9365       Erinaceus        europaeus           hedgehog      FALSE
## 14  9544          Macaca          mulatta            macaque       TRUE
## 15  9593         Gorilla          gorilla            gorilla      FALSE
## 16  9597             Pan         paniscus             bonobo      FALSE
## 17  9598             Pan      troglodytes         chimpanzee      FALSE
## 18  9606            Homo          sapiens              human       TRUE
## 19  9615           Canis lupus familiaris                dog      FALSE
## 20  9685           Felis            catus                cat      FALSE
## 21  9796           Equus         caballus              horse      FALSE
## 22  9823             Sus           scrofa                pig      FALSE
## 23  9913             Bos           taurus             cattle      FALSE
## 24  9986     Oryctolagus        cuniculus             rabbit      FALSE
## 25 10090             Mus         musculus              mouse       TRUE
## 26 10116          Rattus       norvegicus                rat       TRUE
## 27 10141           Cavia        porcellus         guinea pig      FALSE
## 28 13616     Monodelphis        domestica            opossum      FALSE
## 29 28377          Anolis     carolinensis        green anole      FALSE
##       EST IN_SITU RNA_SEQ
## 1  FALSE    TRUE    TRUE
## 2  FALSE   FALSE    TRUE
## 3   TRUE    TRUE    TRUE
## 4  FALSE   FALSE    TRUE
## 5  FALSE   FALSE    TRUE
## 6  FALSE   FALSE    TRUE
## 7  FALSE   FALSE    TRUE
## 8  FALSE   FALSE    TRUE
## 9   TRUE    TRUE    TRUE
## 10  TRUE    TRUE    TRUE
## 11 FALSE   FALSE    TRUE
## 12 FALSE   FALSE    TRUE
## 13 FALSE   FALSE    TRUE
## 14 FALSE   FALSE    TRUE
## 15 FALSE   FALSE    TRUE
## 16 FALSE   FALSE    TRUE
## 17 FALSE   FALSE    TRUE
## 18  TRUE   FALSE    TRUE
## 19 FALSE   FALSE    TRUE
## 20 FALSE   FALSE    TRUE
## 21 FALSE   FALSE    TRUE
```

```
## 22 FALSE   FALSE    TRUE
## 23 FALSE   FALSE    TRUE
## 24 FALSE   FALSE    TRUE
## 25  TRUE    TRUE    TRUE
## 26 FALSE   FALSE    TRUE
## 27 FALSE   FALSE    TRUE
## 28 FALSE   FALSE    TRUE
## 29 FALSE   FALSE    TRUE
```

```
# specify species and data type
# the examples in this paper are based on Bgee release 14.0
# the following line targets the latest Bgee release. In order to target
# specifically the release 14.0, add the parameter 'release="14.0"'
bgee.affymetrix <- Bgee$new(species="Mus_musculus", dataType="affymetrix")
```

```
##
## Querying Bgee to get release information...
##
## Building URL to query species in Bgee release 14_0...
##
## Submitting URL to Bgee webservice... (https://r.bgee.org/?page=r_package&act
ion=get_all_species&display_type=tsv&source=BgeeDB_R_package&source_version=2.6
.2)
##
## Query to Bgee webservice successful!
##
## API key built: fd82223c0f21b0420ddb5ea56f9c9119fae90c2ea687fab7c05a2ab5b92af
45e88e3151f9b01068994a2b2ff7fa7abf3d6eb239add8939046a9ec3279bc16efc
```

```
# retrieve annotation of all mouse affymetrix datasets in Bgee
annotation.bgee.mouse.affymetrix <- getAnnotation(bgee.affymetrix)
```

```
##
## Saved annotation files in /Users/akomljen/Project_Fun_in_R/Mus_musculus_Bgee
_14_0 folder.
```

```
# retrieve annotations of samples and experiments
sample.annotation <- annotation.bgee.mouse.affymetrix$sample.annotation
experiment.annotation <- annotation.bgee.mouse.affymetrix$experiment.annotation

# list experiments including a zygote sample
selected.experiments <- unique(sample.annotation$Experiment.ID[sample.annotatio
n$Stage.ID == "UBERON:0000106"])
experiment.annotation[experiment.annotation$Experiment.ID %in% selected.experim
ents,]
```

```
##     Experiment.ID
## 24        GSE1749
## 32      E-MEXP-51
## 114      GSE18290
##
Experiment.name
## 24                                                             Transcription p
rofiling of mouse embryo to assess gene expression during preimplantation mouse
development
## 32  Transcription profiling of mouse pre-implantation development over twelv
e time points from the germinal vesicle (GV) stage oocyte to the late (expanded
) blastocyst
## 114
Transcription profiling of human, mouse, Bos taurus embryo
##     Chip.count Condition.count Organ.stage.count Organ.count Stage.count
## 24          42               5                 5           4           5
## 32          35               9                 9           5           7
## 114         12               4                 4           3           4
##     Sex.count Strain.count  Data.source
## 24          1            1          GEO
## 32          1            1 ArrayExpress
## 114         0            0          GEO
##                                                  Data.source.URL
## 24   http://www.ncbi.nlm.nih.gov/geo/query/acc.cgi?acc=GSE1749
## 32     http://www.ebi.ac.uk/arrayexpress/experiments/E-MEXP-51
## 114 http://www.ncbi.nlm.nih.gov/geo/query/acc.cgi?acc=GSE18290
##
Bgee.normalized.data.URL
## 24    ftp://ftp.bgee.org/bgee_v14_0/download/processed_expr_values/affymetri
x/Mus_musculus/Mus_musculus_Affymetrix_probesets_GSE1749.tar.gz
## 32  ftp://ftp.bgee.org/bgee_v14_0/download/processed_expr_values/affymetrix/
Mus_musculus/Mus_musculus_Affymetrix_probesets_E-MEXP-51.tar.gz
## 114  ftp://ftp.bgee.org/bgee_v14_0/download/processed_expr_values/affymetrix
/Mus_musculus/Mus_musculus_Affymetrix_probesets_GSE18290.tar.gz
##                                           Bgee.raw.files.URL
## 24   ftp://ftp.bgee.org/affymetrix_data/mas5_files/GSE1749/
## 32  ftp://ftp.bgee.org/affymetrix_data/cel_files/E-MEXP-51/
## 114  ftp://ftp.bgee.org/affymetrix_data/cel_files/GSE18290/
##
Experiment.description
## 24  Studies using low-resolution methods to assess gene expression during pr
eimplantation mouse development indicate that changes in gene expression either
precede or occur concomitantly with the major morphological transitions, that i
s, conversion of the oocyte to totipotent 2-cell blastomeres, compaction, and b
lastocyst formation. Using microarrays, we characterized global changes in gene
expression and used Expression Analysis Systematic Explorer (EASE) to identify
biological and molecular processes that accompany and likely underlie these tra
nsitions. The analysis confirmed previously described processes or events, but
more important, EASE revealed new insights. Response to DNA damage and DNA repa
ir genes are overrepresented in the oocyte compared to 1-cell through blastocys
t stages and may reflect the oocyte's response to selective pressures to insure
genomic integrity; fertilization results in changes in the transcript profile i
```

```
n the 1-cell embryo that are far greater than previously recognized; and genome
activation during 2-cell stage may not be as global and promiscuous as previous
ly proposed, but rather far more selective, with genes involved in transcriptio
n and RNA processing being preferentially expressed. These results validate thi
s hypothesis-generating approach by identifying genes involved in critical biol
ogical processes that can be the subject of a more traditional hypothesis-drive
n approach.
## 32
The goal of the experiments was to profile and analyze gene activity during mur
ine pre-implantation development. Samples were collected at twelve time points
from the germinal vesicle (GV) stage oocyte to the late (expanded) blastocyst.
## 114
The process of early development of mammals is subtly and accurately controlled
by the regulation networks of embryo cells. Time course expression data measure
d at different stages during early embryo development process can give us valua
ble information by revealing the dynamic expression patterns of genes in genome
wide scale. In this study, bovine embryo expression data were generated at oocy
te, one cell stage, two cell stage, four cell stage, eight cell stage, sixteen
cell stage, morula, and blastocyst; Human embryo expression data were generated
at one cell stage, two cell stage, four cell stage, eight cell stage, morula, a
nd blastocyst; Mouse embryo expression data were generated at one cell stage, t
wo cell stage, four cell stage, eight cell stage, morula, and blastocyst. Exper
iment Overall Design: Bovine, Human, and Mouse embryos were harvested at succes
sive stage from oocyte to blastocyste. Total RNAs were extracted, amplified and
hybridized onto Affymetrix microarrays.
```

```
# stages sampled in each of these experiments
unique(sample.annotation[sample.annotation$Experiment.ID %in% selected.experime
nts, c("Experiment.ID", "Stage.name")])
```

```
##      Experiment.ID                Stage.name
## 1536       GSE1749                life cycle
## 1540       GSE1749            blastula stage
## 1543       GSE1749              zygote stage
## 1546       GSE1749  Theiler stage 02 (mouse)
## 1550       GSE1749  Theiler stage 03 (mouse)
## 1836     E-MEXP-51              zygote stage
## 1840     E-MEXP-51 post-juvenile adult stage
## 1847     E-MEXP-51  Theiler stage 03 (mouse)
## 1849     E-MEXP-51  Theiler stage 02 (mouse)
## 1862     E-MEXP-51  Theiler stage 04 (mouse)
## 1866     E-MEXP-51  Theiler stage 05 (mouse)
## 1868     E-MEXP-51  Theiler stage 06 (mouse)
## 3458      GSE18290            blastula stage
## 3460      GSE18290              zygote stage
## 3463      GSE18290  Theiler stage 02 (mouse)
## 3467      GSE18290  Theiler stage 03 (mouse)
```

```
# List all samples from E-MEXP-51 in Bgee
sample.annotation[sample.annotation$Experiment.ID == "E-MEXP-51",]
```

```
##      Experiment.ID         Chip.ID Anatomical.entity.ID
## 1836     E-MEXP-51         Zygote1           CL:0000365
## 1837     E-MEXP-51         Zygote2           CL:0000365
## 1838     E-MEXP-51         Zygote3           CL:0000365
## 1839     E-MEXP-51         Zygote4           CL:0000365
## 1840     E-MEXP-51       GVoocyte1           CL:0000654
## 1841     E-MEXP-51       GVoocyte2           CL:0000654
## 1842     E-MEXP-51       GVoocyte3           CL:0000654
## 1843     E-MEXP-51       GVoocyte4           CL:0000654
## 1844     E-MEXP-51      MIIoocyte1           CL:0000655
## 1845     E-MEXP-51      MIIoocyte2           CL:0000655
## 1846     E-MEXP-51      MIIoocyte3           CL:0000655
## 1847     E-MEXP-51         16cell1       UBERON:0000085
## 1848     E-MEXP-51         16cell2       UBERON:0000085
## 1849     E-MEXP-51    Early2-cell1       UBERON:0000922
## 1850     E-MEXP-51    Early2-cell2       UBERON:0000922
## 1851     E-MEXP-51     Late2-cell1       UBERON:0000922
## 1852     E-MEXP-51     Late2-cell2       UBERON:0000922
## 1853     E-MEXP-51      Mid2-cell1       UBERON:0000922
## 1854     E-MEXP-51      Mid2-cell2       UBERON:0000922
## 1855     E-MEXP-51      Mid2-cell3       UBERON:0000922
## 1856     E-MEXP-51          4Cell1       UBERON:0000922
## 1857     E-MEXP-51          4cell2       UBERON:0000922
## 1858     E-MEXP-51          4cell3       UBERON:0000922
## 1859     E-MEXP-51          8cell1       UBERON:0000922
## 1860     E-MEXP-51          8cell2       UBERON:0000922
## 1861     E-MEXP-51          8cell3       UBERON:0000922
## 1862     E-MEXP-51 EarlyBlastocyst1       UBERON:0000922
## 1863     E-MEXP-51 EarlyBlastocyst2       UBERON:0000922
## 1864     E-MEXP-51 EarlyBlastocyst3       UBERON:0000922
## 1865     E-MEXP-51 EarlyBlastocyst4       UBERON:0000922
## 1866     E-MEXP-51   MidBlastocyst1       UBERON:0000922
## 1867     E-MEXP-51   MidBlastocyst2       UBERON:0000922
## 1868     E-MEXP-51  LateBlastocyst1       UBERON:0000922
## 1869     E-MEXP-51  LateBlastocyst2       UBERON:0000922
## 1870     E-MEXP-51  LateBlastocyst3       UBERON:0000922
##      Anatomical.entity.name       Stage.ID               Stage.name
## 1836                 zygote UBERON:0000106             zygote stage
## 1837                 zygote UBERON:0000106             zygote stage
## 1838                 zygote UBERON:0000106             zygote stage
## 1839                 zygote UBERON:0000106             zygote stage
## 1840         primary oocyte UBERON:0000113 post-juvenile adult stage
## 1841         primary oocyte UBERON:0000113 post-juvenile adult stage
## 1842         primary oocyte UBERON:0000113 post-juvenile adult stage
## 1843         primary oocyte UBERON:0000113 post-juvenile adult stage
## 1844       secondary oocyte UBERON:0000113 post-juvenile adult stage
## 1845       secondary oocyte UBERON:0000113 post-juvenile adult stage
## 1846       secondary oocyte UBERON:0000113 post-juvenile adult stage
## 1847                 morula MmusDv:0000006  Theiler stage 03 (mouse)
## 1848                 morula MmusDv:0000006  Theiler stage 03 (mouse)
## 1849                 embryo MmusDv:0000005  Theiler stage 02 (mouse)
## 1850                 embryo MmusDv:0000005  Theiler stage 02 (mouse)
```

```
## 1851                 embryo MmusDv:0000005  Theiler stage 02 (mouse)
## 1852                 embryo MmusDv:0000005  Theiler stage 02 (mouse)
## 1853                 embryo MmusDv:0000005  Theiler stage 02 (mouse)
## 1854                 embryo MmusDv:0000005  Theiler stage 02 (mouse)
## 1855                 embryo MmusDv:0000005  Theiler stage 02 (mouse)
## 1856                 embryo MmusDv:0000006  Theiler stage 03 (mouse)
## 1857                 embryo MmusDv:0000006  Theiler stage 03 (mouse)
## 1858                 embryo MmusDv:0000006  Theiler stage 03 (mouse)
## 1859                 embryo MmusDv:0000006  Theiler stage 03 (mouse)
## 1860                 embryo MmusDv:0000006  Theiler stage 03 (mouse)
## 1861                 embryo MmusDv:0000006  Theiler stage 03 (mouse)
## 1862                 embryo MmusDv:0000008  Theiler stage 04 (mouse)
## 1863                 embryo MmusDv:0000008  Theiler stage 04 (mouse)
## 1864                 embryo MmusDv:0000008  Theiler stage 04 (mouse)
## 1865                 embryo MmusDv:0000008  Theiler stage 04 (mouse)
## 1866                 embryo MmusDv:0000009  Theiler stage 05 (mouse)
## 1867                 embryo MmusDv:0000009  Theiler stage 05 (mouse)
## 1868                 embryo MmusDv:0000010  Theiler stage 06 (mouse)
## 1869                 embryo MmusDv:0000010  Theiler stage 06 (mouse)
## 1870                 embryo MmusDv:0000010  Theiler stage 06 (mouse)
##                Sex       Strain IQRray.score MAS5.percent.present
## 1836 not annotated C57BL6 x CBA     47883.54                32.01
## 1837 not annotated C57BL6 x CBA     48592.69                33.04
## 1838 not annotated C57BL6 x CBA     46959.46                29.06
## 1839 not annotated C57BL6 x CBA     48053.21                30.73
## 1840        female C57BL6 x CBA     50769.96                33.42
## 1841        female C57BL6 x CBA     51133.57                34.18
## 1842        female C57BL6 x CBA     53766.59                38.92
## 1843        female C57BL6 x CBA     51714.74                38.11
## 1844        female C57BL6 x CBA     47625.00                30.18
## 1845        female C57BL6 x CBA     46662.15                29.14
## 1846        female C57BL6 x CBA     48439.18                31.95
## 1847 not annotated C57BL6 x CBA     54791.02                41.80
## 1848 not annotated C57BL6 x CBA     55477.89                42.30
## 1849 not annotated C57BL6 x CBA     47968.59                34.32
## 1850 not annotated C57BL6 x CBA     47188.40                33.64
## 1851 not annotated C57BL6 x CBA     52613.70                41.14
## 1852 not annotated C57BL6 x CBA     51534.73                40.46
## 1853 not annotated C57BL6 x CBA     50929.56                39.04
## 1854 not annotated C57BL6 x CBA     48914.31                36.87
## 1855 not annotated C57BL6 x CBA     50681.62                39.25
## 1856 not annotated C57BL6 x CBA     54046.04                41.60
## 1857 not annotated C57BL6 x CBA     56463.66                43.79
## 1858 not annotated C57BL6 x CBA     55470.60                42.93
## 1859 not annotated C57BL6 x CBA     49949.18                37.08
## 1860 not annotated C57BL6 x CBA     51584.40                39.13
## 1861 not annotated C57BL6 x CBA     50419.38                35.91
## 1862 not annotated C57BL6 x CBA     53141.50                40.88
## 1863 not annotated C57BL6 x CBA     56462.55                44.11
## 1864 not annotated C57BL6 x CBA     53715.91                42.78
## 1865 not annotated C57BL6 x CBA     54169.92                41.01
## 1866 not annotated C57BL6 x CBA     53501.41                41.45
```

```
## 1867 not annotated C57BL6 x CBA     51745.67                40.51
## 1868 not annotated C57BL6 x CBA     54152.55                43.47
## 1869 not annotated C57BL6 x CBA     53305.39                42.83
## 1870 not annotated C57BL6 x CBA     55872.16                45.41
##      Normalization.type       Scan.date Chip.type.ID  CDF.name
## 1836              gcRMA 02/27/ 16:31:23     A-AFFY-6 MG_U74Av2
## 1837              gcRMA 02/27/ 16:19:35     A-AFFY-6 MG_U74Av2
## 1838              gcRMA 02/20/ 17:56:57     A-AFFY-6 MG_U74Av2
## 1839              gcRMA 02/20/ 18:08:12     A-AFFY-6 MG_U74Av2
## 1840              gcRMA 12/21/ 14:48:27     A-AFFY-6 MG_U74Av2
## 1841              gcRMA 12/21/ 15:00:32     A-AFFY-6 MG_U74Av2
## 1842              gcRMA 12/23/ 15:51:49     A-AFFY-6 MG_U74Av2
## 1843              gcRMA 12/30/ 13:57:52     A-AFFY-6 MG_U74Av2
## 1844              gcRMA 02/20/ 19:22:46     A-AFFY-6 MG_U74Av2
## 1845              gcRMA 02/20/ 19:45:42     A-AFFY-6 MG_U74Av2
## 1846              gcRMA 03/06/ 14:36:39     A-AFFY-6 MG_U74Av2
## 1847              gcRMA 04/26/ 20:26:07     A-AFFY-6 MG_U74Av2
## 1848              gcRMA 04/26/ 19:57:23     A-AFFY-6 MG_U74Av2
## 1849              gcRMA 02/27/ 14:52:08     A-AFFY-6 MG_U74Av2
## 1850              gcRMA 03/06/ 16:20:26     A-AFFY-6 MG_U74Av2
## 1851              gcRMA 12/30/ 14:13:53     A-AFFY-6 MG_U74Av2
## 1852              gcRMA 12/30/ 14:26:59     A-AFFY-6 MG_U74Av2
## 1853              gcRMA 03/14/ 16:51:24     A-AFFY-6 MG_U74Av2
## 1854              gcRMA 03/14/ 16:11:45     A-AFFY-6 MG_U74Av2
## 1855              gcRMA 04/27/ 17:11:34     A-AFFY-6 MG_U74Av2
## 1856              gcRMA 04/25/ 14:33:00     A-AFFY-6 MG_U74Av2
## 1857              gcRMA 04/26/ 20:36:26     A-AFFY-6 MG_U74Av2
## 1858              gcRMA 04/26/ 20:10:43     A-AFFY-6 MG_U74Av2
## 1859              gcRMA 03/06/ 14:22:14     A-AFFY-6 MG_U74Av2
## 1860              gcRMA 03/06/ 16:07:51     A-AFFY-6 MG_U74Av2
## 1861              gcRMA 02/27/ 15:04:39     A-AFFY-6 MG_U74Av2
## 1862              gcRMA 04/26/ 19:33:43     A-AFFY-6 MG_U74Av2
## 1863              gcRMA 04/26/ 19:44:03     A-AFFY-6 MG_U74Av2
## 1864              gcRMA 04/27/ 17:46:32     A-AFFY-6 MG_U74Av2
## 1865              gcRMA 04/25/ 15:03:49     A-AFFY-6 MG_U74Av2
## 1866              gcRMA 04/25/ 15:41:24     A-AFFY-6 MG_U74Av2
## 1867              gcRMA 04/27/ 16:59:27     A-AFFY-6 MG_U74Av2
## 1868              gcRMA 04/27/ 17:24:54     A-AFFY-6 MG_U74Av2
## 1869              gcRMA 04/25/ 14:52:10     A-AFFY-6 MG_U74Av2
## 1870              gcRMA 04/27/ 17:34:59     A-AFFY-6 MG_U74Av2
##                                Chip.type.name
## 1836 Affymetrix GeneChip Murine Genome U74Av2
## 1837 Affymetrix GeneChip Murine Genome U74Av2
## 1838 Affymetrix GeneChip Murine Genome U74Av2
## 1839 Affymetrix GeneChip Murine Genome U74Av2
## 1840 Affymetrix GeneChip Murine Genome U74Av2
## 1841 Affymetrix GeneChip Murine Genome U74Av2
## 1842 Affymetrix GeneChip Murine Genome U74Av2
## 1843 Affymetrix GeneChip Murine Genome U74Av2
## 1844 Affymetrix GeneChip Murine Genome U74Av2
## 1845 Affymetrix GeneChip Murine Genome U74Av2
## 1846 Affymetrix GeneChip Murine Genome U74Av2
```

```
## 1847 Affymetrix GeneChip Murine Genome U74Av2
## 1848 Affymetrix GeneChip Murine Genome U74Av2
## 1849 Affymetrix GeneChip Murine Genome U74Av2
## 1850 Affymetrix GeneChip Murine Genome U74Av2
## 1851 Affymetrix GeneChip Murine Genome U74Av2
## 1852 Affymetrix GeneChip Murine Genome U74Av2
## 1853 Affymetrix GeneChip Murine Genome U74Av2
## 1854 Affymetrix GeneChip Murine Genome U74Av2
## 1855 Affymetrix GeneChip Murine Genome U74Av2
## 1856 Affymetrix GeneChip Murine Genome U74Av2
## 1857 Affymetrix GeneChip Murine Genome U74Av2
## 1858 Affymetrix GeneChip Murine Genome U74Av2
## 1859 Affymetrix GeneChip Murine Genome U74Av2
## 1860 Affymetrix GeneChip Murine Genome U74Av2
## 1861 Affymetrix GeneChip Murine Genome U74Av2
## 1862 Affymetrix GeneChip Murine Genome U74Av2
## 1863 Affymetrix GeneChip Murine Genome U74Av2
## 1864 Affymetrix GeneChip Murine Genome U74Av2
## 1865 Affymetrix GeneChip Murine Genome U74Av2
## 1866 Affymetrix GeneChip Murine Genome U74Av2
## 1867 Affymetrix GeneChip Murine Genome U74Av2
## 1868 Affymetrix GeneChip Murine Genome U74Av2
## 1869 Affymetrix GeneChip Murine Genome U74Av2
## 1870 Affymetrix GeneChip Murine Genome U74Av2
##      IQRray.score.threshold.for.the.chip.type
## 1836                                 44245.73
## 1837                                 44245.73
## 1838                                 44245.73
## 1839                                 44245.73
## 1840                                 44245.73
## 1841                                 44245.73
## 1842                                 44245.73
## 1843                                 44245.73
## 1844                                 44245.73
## 1845                                 44245.73
## 1846                                 44245.73
## 1847                                 44245.73
## 1848                                 44245.73
## 1849                                 44245.73
## 1850                                 44245.73
## 1851                                 44245.73
## 1852                                 44245.73
## 1853                                 44245.73
## 1854                                 44245.73
## 1855                                 44245.73
## 1856                                 44245.73
## 1857                                 44245.73
## 1858                                 44245.73
## 1859                                 44245.73
## 1860                                 44245.73
## 1861                                 44245.73
## 1862                                 44245.73
```

```
## 1863                                  44245.73
## 1864                                  44245.73
## 1865                                  44245.73
## 1866                                  44245.73
## 1867                                  44245.73
## 1868                                  44245.73
## 1869                                  44245.73
## 1870                                  44245.73
##      MAS5.percent.present.threshold.for.the.chip.type  Data.source
## 1836                                             26.68 ArrayExpress
## 1837                                             26.68 ArrayExpress
## 1838                                             26.68 ArrayExpress
## 1839                                             26.68 ArrayExpress
## 1840                                             26.68 ArrayExpress
## 1841                                             26.68 ArrayExpress
## 1842                                             26.68 ArrayExpress
## 1843                                             26.68 ArrayExpress
## 1844                                             26.68 ArrayExpress
## 1845                                             26.68 ArrayExpress
## 1846                                             26.68 ArrayExpress
## 1847                                             26.68 ArrayExpress
## 1848                                             26.68 ArrayExpress
## 1849                                             26.68 ArrayExpress
## 1850                                             26.68 ArrayExpress
## 1851                                             26.68 ArrayExpress
## 1852                                             26.68 ArrayExpress
## 1853                                             26.68 ArrayExpress
## 1854                                             26.68 ArrayExpress
## 1855                                             26.68 ArrayExpress
## 1856                                             26.68 ArrayExpress
## 1857                                             26.68 ArrayExpress
## 1858                                             26.68 ArrayExpress
## 1859                                             26.68 ArrayExpress
## 1860                                             26.68 ArrayExpress
## 1861                                             26.68 ArrayExpress
## 1862                                             26.68 ArrayExpress
## 1863                                             26.68 ArrayExpress
## 1864                                             26.68 ArrayExpress
## 1865                                             26.68 ArrayExpress
## 1866                                             26.68 ArrayExpress
## 1867                                             26.68 ArrayExpress
## 1868                                             26.68 ArrayExpress
## 1869                                             26.68 ArrayExpress
## 1870                                             26.68 ArrayExpress
##      Data.source.URL
## 1836            <NA>
## 1837            <NA>
## 1838            <NA>
## 1839            <NA>
## 1840            <NA>
## 1841            <NA>
## 1842            <NA>
```

```
## 1843            <NA>
## 1844            <NA>
## 1845            <NA>
## 1846            <NA>
## 1847            <NA>
## 1848            <NA>
## 1849            <NA>
## 1850            <NA>
## 1851            <NA>
## 1852            <NA>
## 1853            <NA>
## 1854            <NA>
## 1855            <NA>
## 1856            <NA>
## 1857            <NA>
## 1858            <NA>
## 1859            <NA>
## 1860            <NA>
## 1861            <NA>
## 1862            <NA>
## 1863            <NA>
## 1864            <NA>
## 1865            <NA>
## 1866            <NA>
## 1867            <NA>
## 1868            <NA>
## 1869            <NA>
## 1870            <NA>
##
Bgee.normalized.data.URL
## 1836 ftp://ftp.bgee.org/bgee_v14_0/download/processed_expr_values/affymetrix
/Mus_musculus/Mus_musculus_Affymetrix_probesets_E-MEXP-51.tar.gz
## 1837 ftp://ftp.bgee.org/bgee_v14_0/download/processed_expr_values/affymetrix
/Mus_musculus/Mus_musculus_Affymetrix_probesets_E-MEXP-51.tar.gz
## 1838 ftp://ftp.bgee.org/bgee_v14_0/download/processed_expr_values/affymetrix
/Mus_musculus/Mus_musculus_Affymetrix_probesets_E-MEXP-51.tar.gz
## 1839 ftp://ftp.bgee.org/bgee_v14_0/download/processed_expr_values/affymetrix
/Mus_musculus/Mus_musculus_Affymetrix_probesets_E-MEXP-51.tar.gz
## 1840 ftp://ftp.bgee.org/bgee_v14_0/download/processed_expr_values/affymetrix
/Mus_musculus/Mus_musculus_Affymetrix_probesets_E-MEXP-51.tar.gz
## 1841 ftp://ftp.bgee.org/bgee_v14_0/download/processed_expr_values/affymetrix
/Mus_musculus/Mus_musculus_Affymetrix_probesets_E-MEXP-51.tar.gz
## 1842 ftp://ftp.bgee.org/bgee_v14_0/download/processed_expr_values/affymetrix
/Mus_musculus/Mus_musculus_Affymetrix_probesets_E-MEXP-51.tar.gz
## 1843 ftp://ftp.bgee.org/bgee_v14_0/download/processed_expr_values/affymetrix
/Mus_musculus/Mus_musculus_Affymetrix_probesets_E-MEXP-51.tar.gz
## 1844 ftp://ftp.bgee.org/bgee_v14_0/download/processed_expr_values/affymetrix
/Mus_musculus/Mus_musculus_Affymetrix_probesets_E-MEXP-51.tar.gz
## 1845 ftp://ftp.bgee.org/bgee_v14_0/download/processed_expr_values/affymetrix
/Mus_musculus/Mus_musculus_Affymetrix_probesets_E-MEXP-51.tar.gz
## 1846 ftp://ftp.bgee.org/bgee_v14_0/download/processed_expr_values/affymetrix
/Mus_musculus/Mus_musculus_Affymetrix_probesets_E-MEXP-51.tar.gz
```

```
## 1847 ftp://ftp.bgee.org/bgee_v14_0/download/processed_expr_values/affymetrix
/Mus_musculus/Mus_musculus_Affymetrix_probesets_E-MEXP-51.tar.gz
## 1848 ftp://ftp.bgee.org/bgee_v14_0/download/processed_expr_values/affymetrix
/Mus_musculus/Mus_musculus_Affymetrix_probesets_E-MEXP-51.tar.gz
## 1849 ftp://ftp.bgee.org/bgee_v14_0/download/processed_expr_values/affymetrix
/Mus_musculus/Mus_musculus_Affymetrix_probesets_E-MEXP-51.tar.gz
## 1850 ftp://ftp.bgee.org/bgee_v14_0/download/processed_expr_values/affymetrix
/Mus_musculus/Mus_musculus_Affymetrix_probesets_E-MEXP-51.tar.gz
## 1851 ftp://ftp.bgee.org/bgee_v14_0/download/processed_expr_values/affymetrix
/Mus_musculus/Mus_musculus_Affymetrix_probesets_E-MEXP-51.tar.gz
## 1852 ftp://ftp.bgee.org/bgee_v14_0/download/processed_expr_values/affymetrix
/Mus_musculus/Mus_musculus_Affymetrix_probesets_E-MEXP-51.tar.gz
## 1853 ftp://ftp.bgee.org/bgee_v14_0/download/processed_expr_values/affymetrix
/Mus_musculus/Mus_musculus_Affymetrix_probesets_E-MEXP-51.tar.gz
## 1854 ftp://ftp.bgee.org/bgee_v14_0/download/processed_expr_values/affymetrix
/Mus_musculus/Mus_musculus_Affymetrix_probesets_E-MEXP-51.tar.gz
## 1855 ftp://ftp.bgee.org/bgee_v14_0/download/processed_expr_values/affymetrix
/Mus_musculus/Mus_musculus_Affymetrix_probesets_E-MEXP-51.tar.gz
## 1856 ftp://ftp.bgee.org/bgee_v14_0/download/processed_expr_values/affymetrix
/Mus_musculus/Mus_musculus_Affymetrix_probesets_E-MEXP-51.tar.gz
## 1857 ftp://ftp.bgee.org/bgee_v14_0/download/processed_expr_values/affymetrix
/Mus_musculus/Mus_musculus_Affymetrix_probesets_E-MEXP-51.tar.gz
## 1858 ftp://ftp.bgee.org/bgee_v14_0/download/processed_expr_values/affymetrix
/Mus_musculus/Mus_musculus_Affymetrix_probesets_E-MEXP-51.tar.gz
## 1859 ftp://ftp.bgee.org/bgee_v14_0/download/processed_expr_values/affymetrix
/Mus_musculus/Mus_musculus_Affymetrix_probesets_E-MEXP-51.tar.gz
## 1860 ftp://ftp.bgee.org/bgee_v14_0/download/processed_expr_values/affymetrix
/Mus_musculus/Mus_musculus_Affymetrix_probesets_E-MEXP-51.tar.gz
## 1861 ftp://ftp.bgee.org/bgee_v14_0/download/processed_expr_values/affymetrix
/Mus_musculus/Mus_musculus_Affymetrix_probesets_E-MEXP-51.tar.gz
## 1862 ftp://ftp.bgee.org/bgee_v14_0/download/processed_expr_values/affymetrix
/Mus_musculus/Mus_musculus_Affymetrix_probesets_E-MEXP-51.tar.gz
## 1863 ftp://ftp.bgee.org/bgee_v14_0/download/processed_expr_values/affymetrix
/Mus_musculus/Mus_musculus_Affymetrix_probesets_E-MEXP-51.tar.gz
## 1864 ftp://ftp.bgee.org/bgee_v14_0/download/processed_expr_values/affymetrix
/Mus_musculus/Mus_musculus_Affymetrix_probesets_E-MEXP-51.tar.gz
## 1865 ftp://ftp.bgee.org/bgee_v14_0/download/processed_expr_values/affymetrix
/Mus_musculus/Mus_musculus_Affymetrix_probesets_E-MEXP-51.tar.gz
## 1866 ftp://ftp.bgee.org/bgee_v14_0/download/processed_expr_values/affymetrix
/Mus_musculus/Mus_musculus_Affymetrix_probesets_E-MEXP-51.tar.gz
## 1867 ftp://ftp.bgee.org/bgee_v14_0/download/processed_expr_values/affymetrix
/Mus_musculus/Mus_musculus_Affymetrix_probesets_E-MEXP-51.tar.gz
## 1868 ftp://ftp.bgee.org/bgee_v14_0/download/processed_expr_values/affymetrix
/Mus_musculus/Mus_musculus_Affymetrix_probesets_E-MEXP-51.tar.gz
## 1869 ftp://ftp.bgee.org/bgee_v14_0/download/processed_expr_values/affymetrix
/Mus_musculus/Mus_musculus_Affymetrix_probesets_E-MEXP-51.tar.gz
## 1870 ftp://ftp.bgee.org/bgee_v14_0/download/processed_expr_values/affymetrix
/Mus_musculus/Mus_musculus_Affymetrix_probesets_E-MEXP-51.tar.gz
##                                 Bgee.normalized.data.file
## 1836 Mus_musculus_probesets_E-MEXP-51_A-AFFY-6_gcRMA.tsv
## 1837 Mus_musculus_probesets_E-MEXP-51_A-AFFY-6_gcRMA.tsv
## 1838 Mus_musculus_probesets_E-MEXP-51_A-AFFY-6_gcRMA.tsv
```

```
## 1839 Mus_musculus_probesets_E-MEXP-51_A-AFFY-6_gcRMA.tsv
## 1840 Mus_musculus_probesets_E-MEXP-51_A-AFFY-6_gcRMA.tsv
## 1841 Mus_musculus_probesets_E-MEXP-51_A-AFFY-6_gcRMA.tsv
## 1842 Mus_musculus_probesets_E-MEXP-51_A-AFFY-6_gcRMA.tsv
## 1843 Mus_musculus_probesets_E-MEXP-51_A-AFFY-6_gcRMA.tsv
## 1844 Mus_musculus_probesets_E-MEXP-51_A-AFFY-6_gcRMA.tsv
## 1845 Mus_musculus_probesets_E-MEXP-51_A-AFFY-6_gcRMA.tsv
## 1846 Mus_musculus_probesets_E-MEXP-51_A-AFFY-6_gcRMA.tsv
## 1847 Mus_musculus_probesets_E-MEXP-51_A-AFFY-6_gcRMA.tsv
## 1848 Mus_musculus_probesets_E-MEXP-51_A-AFFY-6_gcRMA.tsv
## 1849 Mus_musculus_probesets_E-MEXP-51_A-AFFY-6_gcRMA.tsv
## 1850 Mus_musculus_probesets_E-MEXP-51_A-AFFY-6_gcRMA.tsv
## 1851 Mus_musculus_probesets_E-MEXP-51_A-AFFY-6_gcRMA.tsv
## 1852 Mus_musculus_probesets_E-MEXP-51_A-AFFY-6_gcRMA.tsv
## 1853 Mus_musculus_probesets_E-MEXP-51_A-AFFY-6_gcRMA.tsv
## 1854 Mus_musculus_probesets_E-MEXP-51_A-AFFY-6_gcRMA.tsv
## 1855 Mus_musculus_probesets_E-MEXP-51_A-AFFY-6_gcRMA.tsv
## 1856 Mus_musculus_probesets_E-MEXP-51_A-AFFY-6_gcRMA.tsv
## 1857 Mus_musculus_probesets_E-MEXP-51_A-AFFY-6_gcRMA.tsv
## 1858 Mus_musculus_probesets_E-MEXP-51_A-AFFY-6_gcRMA.tsv
## 1859 Mus_musculus_probesets_E-MEXP-51_A-AFFY-6_gcRMA.tsv
## 1860 Mus_musculus_probesets_E-MEXP-51_A-AFFY-6_gcRMA.tsv
## 1861 Mus_musculus_probesets_E-MEXP-51_A-AFFY-6_gcRMA.tsv
## 1862 Mus_musculus_probesets_E-MEXP-51_A-AFFY-6_gcRMA.tsv
## 1863 Mus_musculus_probesets_E-MEXP-51_A-AFFY-6_gcRMA.tsv
## 1864 Mus_musculus_probesets_E-MEXP-51_A-AFFY-6_gcRMA.tsv
## 1865 Mus_musculus_probesets_E-MEXP-51_A-AFFY-6_gcRMA.tsv
## 1866 Mus_musculus_probesets_E-MEXP-51_A-AFFY-6_gcRMA.tsv
## 1867 Mus_musculus_probesets_E-MEXP-51_A-AFFY-6_gcRMA.tsv
## 1868 Mus_musculus_probesets_E-MEXP-51_A-AFFY-6_gcRMA.tsv
## 1869 Mus_musculus_probesets_E-MEXP-51_A-AFFY-6_gcRMA.tsv
## 1870 Mus_musculus_probesets_E-MEXP-51_A-AFFY-6_gcRMA.tsv
##                                                                    Bgee.raw.f
ile.URL
## 1836         ftp://ftp.bgee.org/affymetrix_data/cel_files/E-MEXP-51/Zygote1
.CEL.gz
## 1837         ftp://ftp.bgee.org/affymetrix_data/cel_files/E-MEXP-51/Zygote2
.CEL.gz
## 1838         ftp://ftp.bgee.org/affymetrix_data/cel_files/E-MEXP-51/Zygote3
.CEL.gz
## 1839         ftp://ftp.bgee.org/affymetrix_data/cel_files/E-MEXP-51/Zygote4
.CEL.gz
## 1840       ftp://ftp.bgee.org/affymetrix_data/cel_files/E-MEXP-51/GVoocyte1
.CEL.gz
## 1841       ftp://ftp.bgee.org/affymetrix_data/cel_files/E-MEXP-51/GVoocyte2
.CEL.gz
## 1842       ftp://ftp.bgee.org/affymetrix_data/cel_files/E-MEXP-51/GVoocyte3
.CEL.gz
## 1843       ftp://ftp.bgee.org/affymetrix_data/cel_files/E-MEXP-51/GVoocyte4
.CEL.gz
## 1844      ftp://ftp.bgee.org/affymetrix_data/cel_files/E-MEXP-51/MIIoocyte1
.CEL.gz
```

```
## 1845       ftp://ftp.bgee.org/affymetrix_data/cel_files/E-MEXP-51/MIIoocyte2
.CEL.gz
## 1846       ftp://ftp.bgee.org/affymetrix_data/cel_files/E-MEXP-51/MIIoocyte3
.CEL.gz
## 1847          ftp://ftp.bgee.org/affymetrix_data/cel_files/E-MEXP-51/16cell1
.CEL.gz
## 1848          ftp://ftp.bgee.org/affymetrix_data/cel_files/E-MEXP-51/16cell2
.CEL.gz
## 1849     ftp://ftp.bgee.org/affymetrix_data/cel_files/E-MEXP-51/Early2-cell1
.CEL.gz
## 1850     ftp://ftp.bgee.org/affymetrix_data/cel_files/E-MEXP-51/Early2-cell2
.CEL.gz
## 1851      ftp://ftp.bgee.org/affymetrix_data/cel_files/E-MEXP-51/Late2-cell1
.CEL.gz
## 1852      ftp://ftp.bgee.org/affymetrix_data/cel_files/E-MEXP-51/Late2-cell2
.CEL.gz
## 1853       ftp://ftp.bgee.org/affymetrix_data/cel_files/E-MEXP-51/Mid2-cell1
.CEL.gz
## 1854       ftp://ftp.bgee.org/affymetrix_data/cel_files/E-MEXP-51/Mid2-cell2
.CEL.gz
## 1855       ftp://ftp.bgee.org/affymetrix_data/cel_files/E-MEXP-51/Mid2-cell3
.CEL.gz
## 1856           ftp://ftp.bgee.org/affymetrix_data/cel_files/E-MEXP-51/4Cell1
.CEL.gz
## 1857           ftp://ftp.bgee.org/affymetrix_data/cel_files/E-MEXP-51/4cell2
.CEL.gz
## 1858           ftp://ftp.bgee.org/affymetrix_data/cel_files/E-MEXP-51/4cell3
.CEL.gz
## 1859           ftp://ftp.bgee.org/affymetrix_data/cel_files/E-MEXP-51/8cell1
.CEL.gz
## 1860           ftp://ftp.bgee.org/affymetrix_data/cel_files/E-MEXP-51/8cell2
.CEL.gz
## 1861           ftp://ftp.bgee.org/affymetrix_data/cel_files/E-MEXP-51/8cell3
.CEL.gz
## 1862 ftp://ftp.bgee.org/affymetrix_data/cel_files/E-MEXP-51/EarlyBlastocyst1
.CEL.gz
## 1863 ftp://ftp.bgee.org/affymetrix_data/cel_files/E-MEXP-51/EarlyBlastocyst2
.CEL.gz
## 1864 ftp://ftp.bgee.org/affymetrix_data/cel_files/E-MEXP-51/EarlyBlastocyst3
.CEL.gz
## 1865 ftp://ftp.bgee.org/affymetrix_data/cel_files/E-MEXP-51/EarlyBlastocyst4
.CEL.gz
## 1866   ftp://ftp.bgee.org/affymetrix_data/cel_files/E-MEXP-51/MidBlastocyst1
.CEL.gz
## 1867   ftp://ftp.bgee.org/affymetrix_data/cel_files/E-MEXP-51/MidBlastocyst2
.CEL.gz
## 1868  ftp://ftp.bgee.org/affymetrix_data/cel_files/E-MEXP-51/LateBlastocyst1
.CEL.gz
## 1869  ftp://ftp.bgee.org/affymetrix_data/cel_files/E-MEXP-51/LateBlastocyst2
.CEL.gz
## 1870  ftp://ftp.bgee.org/affymetrix_data/cel_files/E-MEXP-51/LateBlastocyst3
.CEL.gz
```

```r
data.E.MEXP.51 <- getData(bgee.affymetrix, experimentId="E-MEXP-51")
```

```
##
## Downloading expression data for the experiment E-MEXP-51 ...
##
## Saved expression data file in /Users/akomljen/Project_Fun_in_R/Mus_musculus_
Bgee_14_0 folder. Now untar /Users/akomljen/Project_Fun_in_R/Mus_musculus_Bgee_
14_0/Mus_musculus_Affymetrix_probesets_E-MEXP-51.tar.gz file...
##
## Saving all data in .rds file...
```

```r
head(data.E.MEXP.51)
```

```
##   Experiment.ID Chip.ID Probeset.ID            Gene.ID
## 1     E-MEXP-51 Zygote1    94814_at ENSMUSG00000000001
## 2     E-MEXP-51 Zygote1    94632_at ENSMUSG00000000003
## 3     E-MEXP-51 Zygote1   102403_at ENSMUSG00000000028
## 4     E-MEXP-51 Zygote1    93028_at ENSMUSG00000000031
## 5     E-MEXP-51 Zygote1    94318_at ENSMUSG00000000049
## 6     E-MEXP-51 Zygote1    98083_at ENSMUSG00000000078
##   Anatomical.entity.ID Anatomical.entity.name       Stage.ID   Stage.name
## 1           CL:0000365                 zygote UBERON:0000106 zygote stage
## 2           CL:0000365                 zygote UBERON:0000106 zygote stage
## 3           CL:0000365                 zygote UBERON:0000106 zygote stage
## 4           CL:0000365                 zygote UBERON:0000106 zygote stage
## 5           CL:0000365                 zygote UBERON:0000106 zygote stage
## 6           CL:0000365                 zygote UBERON:0000106 zygote stage
##             Sex       Strain Log.of.normalized.signal.intensity
## 1 not annotated C57BL6 x CBA                           13.45989
## 2 not annotated C57BL6 x CBA                            2.06161
## 3 not annotated C57BL6 x CBA                           11.49522
## 4 not annotated C57BL6 x CBA                            2.06161
## 5 not annotated C57BL6 x CBA                            2.06161
## 6 not annotated C57BL6 x CBA                            2.06161
##   Detection.flag Detection.quality  State.in.Bgee
## 1        present      high quality Part of a call
## 2         absent      high quality Part of a call
## 3        present      high quality Part of a call
## 4         absent      high quality Part of a call
## 5         absent      high quality Part of a call
## 6         absent      high quality Part of a call
```

```r
data.E.MEXP.51.formatted <- formatData(bgee.affymetrix,
                                       data.E.MEXP.51,
                                       callType="all",
                                       stats="intensities")
```

```
##
## Extracting expression data matrix...
##
## Extracting features information...
##
## Extracting samples information...
```

```
data.E.MEXP.51.formatted
```

```
## ExpressionSet (storageMode: lockedEnvironment)
## assayData: 8954 features, 35 samples
##   element names: exprs
## protocolData: none
## phenoData
##   sampleNames: 16cell1 16cell2 ... Zygote4 (35 total)
##   varLabels: Chip.ID Anatomical.entity.ID ... Stage.name (5 total)
##   varMetadata: labelDescription
## featureData
##   featureNames: 100001_at 100002_at ...
##     AFFX-TransRecMur/X57349_M_at (8954 total)
##   fvarLabels: Probeset.ID Gene.ID
##   fvarMetadata: labelDescription
## experimentData: use 'experimentData(object)'
## Annotation:
```

```
# matrix of expression intensities
head(exprs(data.E.MEXP.51.formatted))
```

```
##           16cell1 16cell2  4Cell1  4cell2  4cell3  8cell1  8cell2  8cell3
## 100001_at 2.06161 2.06161 2.06161 2.06161 2.06161 2.06161 2.06161 2.21218
## 100002_at 2.06161 2.06161 2.06161 2.06161 2.06161 2.06161 2.06161 2.06161
## 100003_at 2.24806 2.06161 2.87344 4.08819 2.08289 2.06161 2.28084 2.06161
## 100004_at 6.47901 6.93907 5.68899 5.68262 5.76980 7.40950 7.41287 6.15540
## 100005_at 4.84777 4.84518 4.84777 4.84777 4.84777 4.46738 4.83575 4.84296
## 100006_at 2.06161 2.06161 2.06161 2.06161 2.06161 2.06161 2.06161 2.06161
##           Early2-cell1 Early2-cell2 EarlyBlastocyst1 EarlyBlastocyst2
## 100001_at      2.06161      2.06161          2.06161          2.06161
## 100002_at      2.06161      2.06161          2.06161          2.06161
## 100003_at      2.06161      2.06161          2.06161          2.06161
## 100004_at      5.63148      5.68254          6.75183          6.43474
## 100005_at      4.84518      4.84777          4.84965          4.84777
## 100006_at      2.06161      2.06161          2.06161          2.06161
##           EarlyBlastocyst3 EarlyBlastocyst4 GVoocyte1 GVoocyte2 GVoocyte3
## 100001_at          2.06161          2.06161   2.06161   2.06161   2.06161
## 100002_at          2.06161          2.06161   2.06161   2.06161   2.06161
## 100003_at          2.44777          2.06161   2.06161   2.06161   2.06161
## 100004_at          6.34930          5.68269   5.68254   4.22671   3.63535
## 100005_at          4.84777          4.84777   4.84777   4.84777   5.22159
## 100006_at          2.06161          2.06161   2.06161   2.06161   2.06161
##           GVoocyte4 Late2-cell1 Late2-cell2 LateBlastocyst1
## 100001_at   2.06161     2.06161     2.06161         2.06161
## 100002_at   2.06161     2.06161     2.06161         2.06161
## 100003_at   2.06161     2.06161     2.06161         2.06165
## 100004_at   4.24684     5.68262     5.68262         6.65076
## 100005_at   4.84777     5.29413     5.15765         4.84777
## 100006_at   2.06161     2.06161     2.06161         2.06161
##           LateBlastocyst2 LateBlastocyst3 Mid2-cell1 Mid2-cell2 Mid2-cell3
## 100001_at         2.06161         2.06161    2.06161    2.06161    2.06161
## 100002_at         2.06161         2.06161    2.06161    2.06161    2.06161
## 100003_at         2.08678         2.78037    2.06161    2.06161    2.06161
## 100004_at         5.68262         6.52297    5.68254    5.42192    5.68254
## 100005_at         4.84518         4.84777    7.36354    4.84777    6.46559
## 100006_at         2.06161         2.06161    2.06161    2.06161    2.06161
##           MidBlastocyst1 MidBlastocyst2 MIIoocyte1 MIIoocyte2 MIIoocyte3
## 100001_at        2.06161        2.06161    2.06161    2.06161    2.06161
## 100002_at        2.06161        2.06161    2.06161    2.06161    2.06161
## 100003_at        2.06161        2.06161    2.06161    2.06161    2.06161
## 100004_at        6.21385        5.68262    5.68262    5.68254    5.68254
## 100005_at        4.82564        4.84777    4.84777    4.84777    4.85231
## 100006_at        2.06161        2.06161    2.06161    2.06161    2.06161
##           Zygote1 Zygote2 Zygote3 Zygote4
## 100001_at 2.06161 2.06161 2.06161 2.06161
## 100002_at 2.06161 2.06161 2.06161 2.06161
## 100003_at 2.06161 2.06161 2.06161 2.06161
## 100004_at 5.62915 5.68254 5.68262 5.02320
## 100005_at 4.84775 4.84777 4.84777 6.69624
## 100006_at 2.06161 2.06161 2.06161 2.06161
```

```
# annotation of samples
pData(data.E.MEXP.51.formatted)
```

```
##                            Chip.ID Anatomical.entity.ID
## 16cell1                    16cell1       UBERON:0000085
## 16cell2                    16cell2       UBERON:0000085
## 4Cell1                      4Cell1       UBERON:0000922
## 4cell2                      4cell2       UBERON:0000922
## 4cell3                      4cell3       UBERON:0000922
## 8cell1                      8cell1       UBERON:0000922
## 8cell2                      8cell2       UBERON:0000922
## 8cell3                      8cell3       UBERON:0000922
## Early2-cell1          Early2-cell1       UBERON:0000922
## Early2-cell2          Early2-cell2       UBERON:0000922
## EarlyBlastocyst1 EarlyBlastocyst1       UBERON:0000922
## EarlyBlastocyst2 EarlyBlastocyst2       UBERON:0000922
## EarlyBlastocyst3 EarlyBlastocyst3       UBERON:0000922
## EarlyBlastocyst4 EarlyBlastocyst4       UBERON:0000922
## GVoocyte1                GVoocyte1           CL:0000654
## GVoocyte2                GVoocyte2           CL:0000654
## GVoocyte3                GVoocyte3           CL:0000654
## GVoocyte4                GVoocyte4           CL:0000654
## Late2-cell1            Late2-cell1       UBERON:0000922
## Late2-cell2            Late2-cell2       UBERON:0000922
## LateBlastocyst1    LateBlastocyst1       UBERON:0000922
## LateBlastocyst2    LateBlastocyst2       UBERON:0000922
## LateBlastocyst3    LateBlastocyst3       UBERON:0000922
## Mid2-cell1              Mid2-cell1       UBERON:0000922
## Mid2-cell2              Mid2-cell2       UBERON:0000922
## Mid2-cell3              Mid2-cell3       UBERON:0000922
## MidBlastocyst1      MidBlastocyst1       UBERON:0000922
## MidBlastocyst2      MidBlastocyst2       UBERON:0000922
## MIIoocyte1              MIIoocyte1           CL:0000655
## MIIoocyte2              MIIoocyte2           CL:0000655
## MIIoocyte3              MIIoocyte3           CL:0000655
## Zygote1                    Zygote1           CL:0000365
## Zygote2                    Zygote2           CL:0000365
## Zygote3                    Zygote3           CL:0000365
## Zygote4                    Zygote4           CL:0000365
##                  Anatomical.entity.name       Stage.ID
## 16cell1                         morula MmusDv:0000006
## 16cell2                         morula MmusDv:0000006
## 4Cell1                          embryo MmusDv:0000006
## 4cell2                          embryo MmusDv:0000006
## 4cell3                          embryo MmusDv:0000006
## 8cell1                          embryo MmusDv:0000006
## 8cell2                          embryo MmusDv:0000006
## 8cell3                          embryo MmusDv:0000006
## Early2-cell1                    embryo MmusDv:0000005
## Early2-cell2                    embryo MmusDv:0000005
## EarlyBlastocyst1                embryo MmusDv:0000008
## EarlyBlastocyst2                embryo MmusDv:0000008
## EarlyBlastocyst3                embryo MmusDv:0000008
## EarlyBlastocyst4                embryo MmusDv:0000008
## GVoocyte1               primary oocyte UBERON:0000113
```

```
## GVoocyte2                primary oocyte UBERON:0000113
## GVoocyte3                primary oocyte UBERON:0000113
## GVoocyte4                primary oocyte UBERON:0000113
## Late2-cell1                      embryo MmusDv:0000005
## Late2-cell2                      embryo MmusDv:0000005
## LateBlastocyst1                  embryo MmusDv:0000010
## LateBlastocyst2                  embryo MmusDv:0000010
## LateBlastocyst3                  embryo MmusDv:0000010
## Mid2-cell1                       embryo MmusDv:0000005
## Mid2-cell2                       embryo MmusDv:0000005
## Mid2-cell3                       embryo MmusDv:0000005
## MidBlastocyst1                   embryo MmusDv:0000009
## MidBlastocyst2                   embryo MmusDv:0000009
## MIIoocyte1             secondary oocyte UBERON:0000113
## MIIoocyte2             secondary oocyte UBERON:0000113
## MIIoocyte3             secondary oocyte UBERON:0000113
## Zygote1                          zygote UBERON:0000106
## Zygote2                          zygote UBERON:0000106
## Zygote3                          zygote UBERON:0000106
## Zygote4                          zygote UBERON:0000106
##                                Stage.name
## 16cell1           Theiler stage 03 (mouse)
## 16cell2           Theiler stage 03 (mouse)
## 4Cell1            Theiler stage 03 (mouse)
## 4cell2            Theiler stage 03 (mouse)
## 4cell3            Theiler stage 03 (mouse)
## 8cell1            Theiler stage 03 (mouse)
## 8cell2            Theiler stage 03 (mouse)
## 8cell3            Theiler stage 03 (mouse)
## Early2-cell1      Theiler stage 02 (mouse)
## Early2-cell2      Theiler stage 02 (mouse)
## EarlyBlastocyst1  Theiler stage 04 (mouse)
## EarlyBlastocyst2  Theiler stage 04 (mouse)
## EarlyBlastocyst3  Theiler stage 04 (mouse)
## EarlyBlastocyst4  Theiler stage 04 (mouse)
## GVoocyte1        post-juvenile adult stage
## GVoocyte2        post-juvenile adult stage
## GVoocyte3        post-juvenile adult stage
## GVoocyte4        post-juvenile adult stage
## Late2-cell1       Theiler stage 02 (mouse)
## Late2-cell2       Theiler stage 02 (mouse)
## LateBlastocyst1   Theiler stage 06 (mouse)
## LateBlastocyst2   Theiler stage 06 (mouse)
## LateBlastocyst3   Theiler stage 06 (mouse)
## Mid2-cell1        Theiler stage 02 (mouse)
## Mid2-cell2        Theiler stage 02 (mouse)
## Mid2-cell3        Theiler stage 02 (mouse)
## MidBlastocyst1    Theiler stage 05 (mouse)
## MidBlastocyst2    Theiler stage 05 (mouse)
## MIIoocyte1       post-juvenile adult stage
## MIIoocyte2       post-juvenile adult stage
## MIIoocyte3       post-juvenile adult stage
```

```
## Zygote1                      zygote stage
## Zygote2                      zygote stage
## Zygote3                      zygote stage
## Zygote4                      zygote stage
```

```
# annotation of probesets
head(fData(data.E.MEXP.51.formatted))
```

```
##            Probeset.ID            Gene.ID
## 100001_at   100001_at ENSMUSG00000002033
## 100002_at   100002_at ENSMUSG00000006522
## 100003_at   100003_at ENSMUSG00000030592
## 100004_at   100004_at ENSMUSG00000037461
## 100005_at   100005_at ENSMUSG00000017386
## 100006_at   100006_at ENSMUSG00000031673
```

```
# specify species and data type
# the examples in this paper are based on Bgee release 14.0
# the following line targets the latest Bgee release. In order to target
# specifically the release 14.0, add the parameter 'release="14.0"'
bgee.rnaseq <- Bgee$new(species="Macaca_mulatta", dataType="rna_seq")
```

```
##
## Querying Bgee to get release information...
##
## NOTE: the file describing Bgee species information for release 14_0 was foun
d in the download directory /Users/akomljen/Project_Fun_in_R. Data will not be
redownloaded.
##
## API key built: fd82223c0f21b0420ddb5ea56f9c9119fae90c2ea687fab7c05a2ab5b92af
45e88e3151f9b01068994a2b2ff7fa7abf3d6eb239add8939046a9ec3279bc16efc
```

```
# retrieve annotations of RNA-seq samples and experiments
annotation.bgee.macaque.rna.seq <- getAnnotation(bgee.rnaseq)
```

```
##
## Saved annotation files in /Users/akomljen/Project_Fun_in_R/Macaca_mulatta_Bg
ee_14_0 folder.
```

```
sample.annotation <- annotation.bgee.macaque.rna.seq$sample.annotation
experiment.annotation <- annotation.bgee.macaque.rna.seq$experiment.annotation

# list experiments including both brain and liver samples
selected.experiments <- intersect(
    unique(
    sample.annotation$Experiment.ID[sample.annotation$Anatomical.entity.ID == "
UBERON:0000955"]),
    unique(
    sample.annotation$Experiment.ID[sample.annotation$Anatomical.entity.ID == "
UBERON:0002107"])
    )
experiment.annotation[experiment.annotation$Experiment.ID %in% selected.experim
ents,]
```

```
##   Experiment.ID
## 2      GSE41637
## 3      GSE30352
##                                                              Experiment.name
## 2 Evolutionary dynamics of gene and isoform regulation in mammalian tissues
## 3              The evolution of gene expression levels in mammalian organs
##   Library.count Condition.count Organ.stage.count Organ.count Stage.count
## 2            27               9                 9           9           1
## 3            13              12                 8           7           3
##   Sex.count Strain.count Data.source
## 2         1            6         GEO
## 3         2            2         GEO
##                                                Data.source.URL
## 2 http://www.ncbi.nlm.nih.gov/geo/query/acc.cgi?acc=GSE41637
## 3 http://www.ncbi.nlm.nih.gov/geo/query/acc.cgi?acc=GSE30352
##
Bgee.normalized.data.URL
## 2 ftp://ftp.bgee.org/bgee_v14_0/download/processed_expr_values/rna_seq/Macac
a_mulatta/Macaca_mulatta_RNA-Seq_read_counts_TPM_FPKM_GSE41637.tsv.tar.gz
## 3 ftp://ftp.bgee.org/bgee_v14_0/download/processed_expr_values/rna_seq/Macac
a_mulatta/Macaca_mulatta_RNA-Seq_read_counts_TPM_FPKM_GSE30352.tsv.tar.gz
##
Experiment.description
## 2
Most mammalian genes produce multiple distinct mRNAs through alternative splici
ng, but the extent of splicing conservation is not clear. To assess tissue-spec
ific transcriptome variation across mammals, we sequenced cDNA from 9 tissues f
rom 4 mammals and one bird in biological triplicate, at unprecedented depth. We
find that while tissue-specific gene expression programs are largely conserved,
alternative splicing is well conserved in only a subset of tissues and is frequ
ently lineage-specific. Thousands of novel, lineage-specific and conserved alte
rnative exons were identified; widely conserved alternative exons had signature
s of binding by MBNL, PTB, RBFOX, STAR and TIA family splicing factors, implica
ting them as ancestral mammalian splicing regulators. Our data also indicates t
hat alternative splicing is often used to alter protein phosphorylatability, de
limiting the scope of kinase signaling.
## 3 Changes in gene expression are thought to underlie many of the phenotypic
differences between species. However, large-scale analyses of gene expression e
volution were until recently prevented by technological limitations. Here we re
port the sequencing of polyadenylated RNA from six organs across ten species th
at represent all major mammalian lineages (placentals, marsupials and monotreme
s) and birds (the evolutionary outgroup), with the goal of understanding the dy
namics of mammalian transcriptome evolution. We show that the rate of gene expr
ession evolution varies among organs, lineages and chromosomes, owing to differ
ences in selective pressures: transcriptome change was slow in nervous tissues
and rapid in testes, slower in rodents than in apes and monotremes, and rapid f
or the X chromosome right after its formation. Although gene expression evoluti
on in mammals was strongly shaped by purifying selection, we identify numerous
potentially selectively driven expression switches, which occurred at different
rates across lineages and tissues and which probably contributed to the specifi
c organ biology of various mammals. Our transcriptome data provide a valuable r
esource for functional and evolutionary analyses of mammalian genomes.
```

```r
# check whether experiments include biological replicates
sample.annotation[sample.annotation$Experiment.ID %in% selected.experiments & (
sample.annotation$Anatomical.entity.ID == "UBERON:0000955" | sample.annotation$
Anatomical.entity.ID == "UBERON:0002107"), c("Experiment.ID",
                      "Library.ID",
                      "Anatomical.entity.ID",
                      "Anatomical.entity.name",
                      "Stage.ID")]
```

```
##    Experiment.ID Library.ID Anatomical.entity.ID Anatomical.entity.name
## 58      GSE41637  SRX196317       UBERON:0000955                  brain
## 59      GSE41637  SRX196326       UBERON:0000955                  brain
## 60      GSE41637  SRX196335       UBERON:0000955                  brain
## 73      GSE41637  SRX196321       UBERON:0002107                  liver
## 74      GSE41637  SRX196330       UBERON:0002107                  liver
## 75      GSE41637  SRX196339       UBERON:0002107                  liver
## 83      GSE30352  SRX081922       UBERON:0000955                  brain
## 84      GSE30352  SRX081923       UBERON:0000955                  brain
## 87      GSE30352  SRX081931       UBERON:0002107                  liver
## 88      GSE30352  SRX081932       UBERON:0002107                  liver
##          Stage.ID
## 58 UBERON:0018241
## 59 UBERON:0018241
## 60 UBERON:0018241
## 73 UBERON:0018241
## 74 UBERON:0018241
## 75 UBERON:0018241
## 83 MmulDv:0000028
## 84 MmulDv:0000028
## 87 MmulDv:0000028
## 88 MmulDv:0000028
```

```r
data.GSE41637 <- getData(bgee.rnaseq, experimentId="GSE41637")
```

```
##
## Downloading expression data for the experiment GSE41637 ...
##
## Saved expression data file in /Users/akomljen/Project_Fun_in_R/Macaca_mulatt
a_Bgee_14_0 folder. Now untar /Users/akomljen/Project_Fun_in_R/Macaca_mulatta_B
gee_14_0/Macaca_mulatta_RNA-Seq_read_counts_TPM_FPKM_GSE41637.tsv.tar.gz file..
.
##
## Saving all data in .rds file...
```

```r
data.GSE41637.formatted <- formatData(bgee.rnaseq, data.GSE41637, callType="all
", stats="counts")
```

```
##
## Extracting expression data matrix...
##
## Extracting features information...
##
## Extracting samples information...
```

```
data.GSE41637.formatted
```

```
## ExpressionSet (storageMode: lockedEnvironment)
## assayData: 30246 features, 27 samples
##   element names: exprs
## protocolData: none
## phenoData
##   sampleNames: SRX196317 SRX196318 ... SRX196343 (27 total)
##   varLabels: Library.ID Anatomical.entity.ID ... Stage.name (5
##     total)
##   varMetadata: labelDescription
## featureData
##   featureNames: ENSMMUG00000000001 ENSMMUG00000000002 ...
##     ENSMMUG00000037447 (30246 total)
##   fvarLabels: Gene.ID
##   fvarMetadata: labelDescription
## experimentData: use 'experimentData(object)'
## Annotation:
```

```
# for simplicity, keep only one sample per condition
data.E.MEXP.51.formatted <- data.E.MEXP.51.formatted[, !duplicated(pData(data.E
.MEXP.51.formatted)[
    c("Anatomical.entity.ID",
      "Anatomical.entity.name",
      "Stage.ID", "Stage.name")])]

# order developmental stages
stages <- c("GVoocyte1","MIIoocyte1","Zygote1",
            "Early2-cell1","4Cell1","16cell1",
            "EarlyBlastocyst1","MidBlastocyst1","LateBlastocyst1")
data.E.MEXP.51.formatted <- data.E.MEXP.51.formatted[, stages]
# filter out rows with no variance
data.E.MEXP.51.formatted <-
data.E.MEXP.51.formatted [apply(exprs(data.E.MEXP.51.formatted), 1, sd) != 0, ]
```

```
# Mfuzz clustering
library(Mfuzz)
```

```
## Loading required package: e1071
```

```
##
## Attaching package: 'DynDoc'
```

```
## The following object is masked from 'package:BiocGenerics':
##
##     path
```

```
# standardize matric of expression data
z.mat <- standardise(data.E.MEXP.51.formatted)
# cluster data into 16 clusters
clusters <- mfuzz(z.mat, centers=16, m=1.25)
```

```
# visualizing clusters
mfuzz.plot2(z.mat,
            cl=clusters,
            mfrow=c(4,4),
            colo="fancy",
            time.labels=row.names(pData(z.mat)),
            las=2, xlab="", ylab="Standardized expression level",
            x11 = FALSE)
```

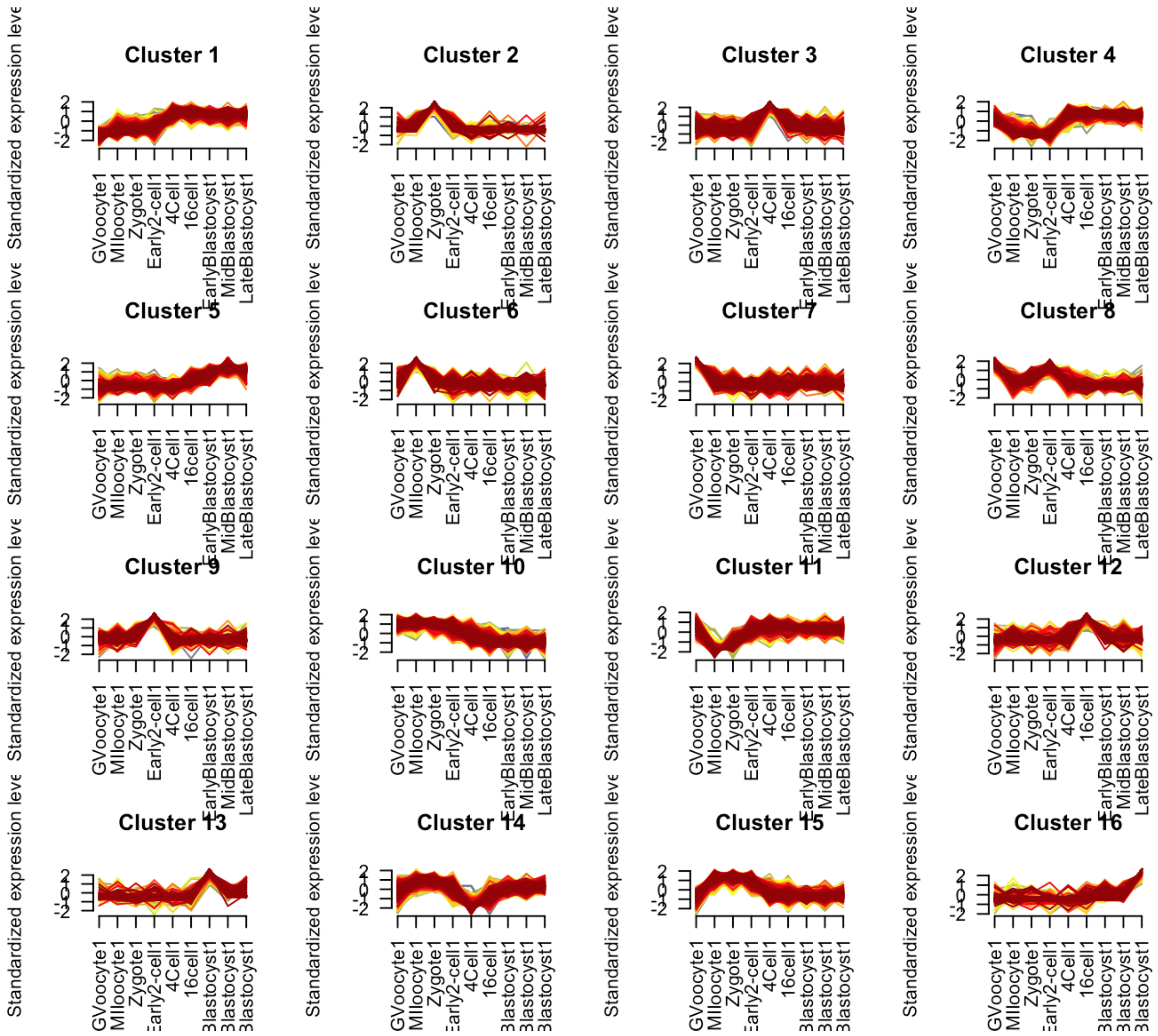


```
# differential expression analysis with edgeR
library(edgeR)
```

```
## Loading required package: limma
```

```
##
## Attaching package: 'limma'
```

```
## The following object is masked from 'package:BiocGenerics':
##
##     plotMA
```

```
# subset the dataset to brain and liver
brain.liver <- data.GSE41637.formatted[, pData(data.GSE41637.formatted)$Anatomi
cal.entity.name %in% c("brain", "liver")]

# filter out very lowly expressed genes
brain.liver.filtered <- brain.liver[rowSums(cpm(brain.liver) > 1) > 3, ]

# create edgeR DGElist object
dge <- DGEList(counts= brain.liver.filtered, group=pData(brain.liver.filtered)$
Anatomical.entity.name)
dge <- calcNormFactors(dge)
dge <- estimateCommonDisp(dge)
dge <- estimateTagwiseDisp(dge)
de <- exactTest(dge, pair=c("brain","liver"))
de.genes <- topTags(de, n=nrow(de))$table
```

```
# MA plot with DE genes highlighted
plotSmear(dge, de.tags=rownames(de.genes)[de.genes$FDR < 0.01], cex=0.3)
```

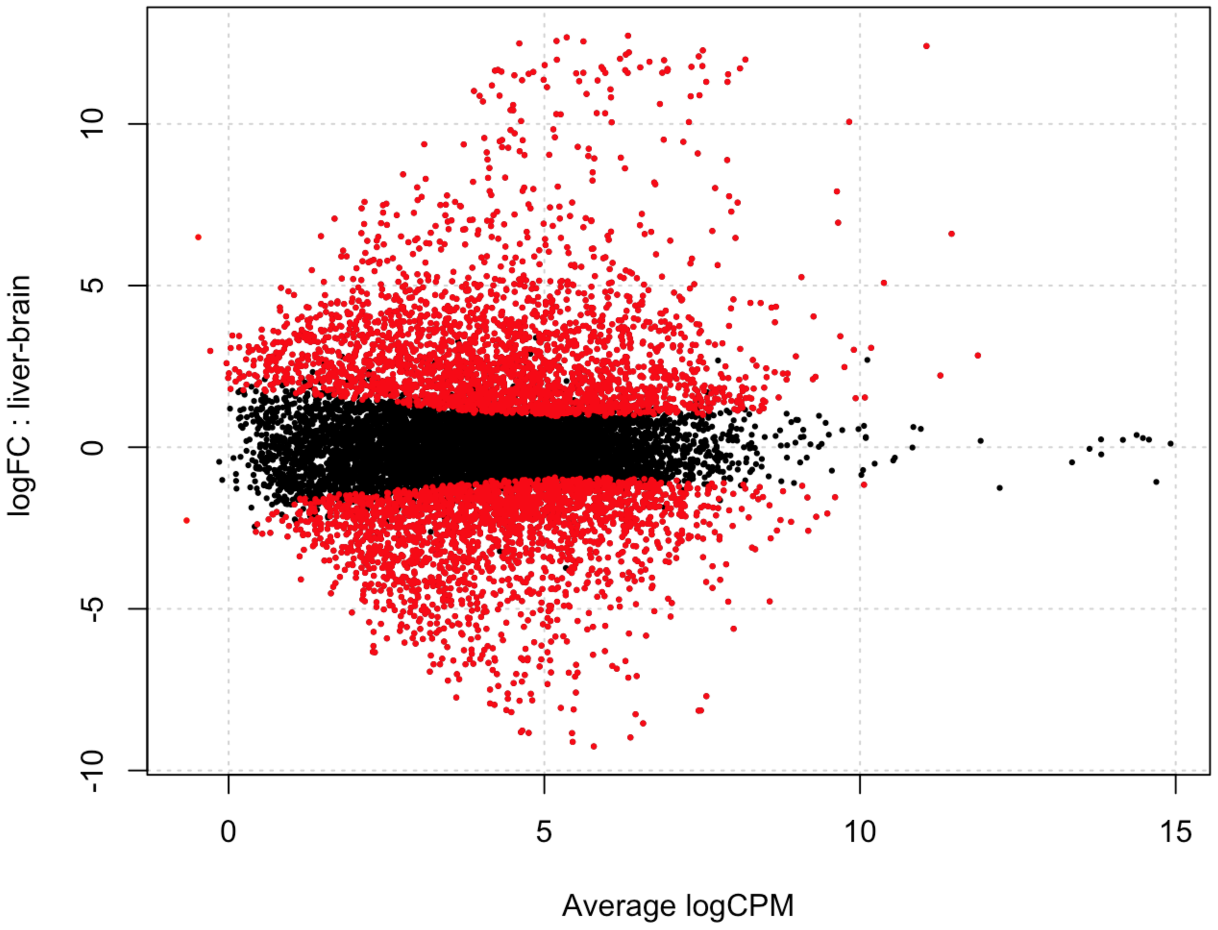

```r
# the examples in this paper are based on Bgee release 14.0
# the following line targets the latest Bgee release. In order to target
# specifically the release 14.0, add the parameter 'release="14.0"'
bgee.topanat <- Bgee$new(species="Danio_rerio")
```

```
##
## NOTE: You did not specify any data type. The argument dataType will be set t
o c("rna_seq","affymetrix","est","in_situ") for the next steps.
##
## Querying Bgee to get release information...
##
## NOTE: the file describing Bgee species information for release 14_0 was foun
d in the download directory /Users/akomljen/Project_Fun_in_R. Data will not be
redownloaded.
##
## API key built: fd82223c0f21b0420ddb5ea56f9c9119fae90c2ea687fab7c05a2ab5b92af
45e88e3151f9b01068994a2b2ff7fa7abf3d6eb239add8939046a9ec3279bc16efc
```

```r
top.anat.data <- loadTopAnatData(bgee.topanat)
```

```
##
## Building URLs to retrieve organ relationships from Bgee.........
##    URL successfully built (https://r.bgee.org/?page=r_package&action=get_ana
t_entity_relations&display_type=tsv&species_list=7955&attr_list=SOURCE_ID&attr_
list=TARGET_ID&api_key=fd82223c0f21b0420ddb5ea56f9c9119fae90c2ea687fab7c05a2ab5
b92af45e88e3151f9b01068994a2b2ff7fa7abf3d6eb239add8939046a9ec3279bc16efc&source
=BgeeDB_R_package&source_version=2.6.2)
##    Submitting URL to Bgee webservice (can be long)
##    Got results from Bgee webservice. Files are written in "/Users/akomljen/P
roject_Fun_in_R/Danio_rerio_Bgee_14_0"
##
## Building URLs to retrieve organ names from Bgee.................
##    URL successfully built (https://r.bgee.org/?page=r_package&action=get_ana
t_entities&display_type=tsv&species_list=7955&attr_list=ID&attr_list=NAME&api_k
ey=fd82223c0f21b0420ddb5ea56f9c9119fae90c2ea687fab7c05a2ab5b92af45e88e3151f9b01
068994a2b2ff7fa7abf3d6eb239add8939046a9ec3279bc16efc&source=BgeeDB_R_package&so
urce_version=2.6.2)
##    Submitting URL to Bgee webservice (can be long)
##    Got results from Bgee webservice. Files are written in "/Users/akomljen/P
roject_Fun_in_R/Danio_rerio_Bgee_14_0"
##
## Building URLs to retrieve mapping of gene to organs from Bgee...
##    URL successfully built (https://r.bgee.org/?page=r_package&action=get_exp
ression_calls&display_type=tsv&species_list=7955&attr_list=GENE_ID&attr_list=AN
AT_ENTITY_ID&api_key=fd82223c0f21b0420ddb5ea56f9c9119fae90c2ea687fab7c05a2ab5b9
2af45e88e3151f9b01068994a2b2ff7fa7abf3d6eb239add8939046a9ec3279bc16efc&source=B
geeDB_R_package&source_version=2.6.2&data_qual=SILVER)
##    Submitting URL to Bgee webservice (can be long)
##    Got results from Bgee webservice. Files are written in "/Users/akomljen/P
roject_Fun_in_R/Danio_rerio_Bgee_14_0"
##
## Parsing the results.........................................
##
## Adding BGEE:0 as unique root of all terms of the ontology.......
##
## Done.
```

```r
library(biomaRt)

# zebrafish data in Ensembl 84 (stable link)
ensembl <- useMart("ENSEMBL_MART_ENSEMBL",
                   dataset="drerio_gene_ensembl",
                   host="mar2016.archive.ensembl.org")

# get the mapping of Ensembl genes to phenotypes
genes.to.phenotypes <- getBM(filters=c("phenotype_source"),
                             value=c("ZFIN"),
                             attributes=c("ensembl_gene_id","phenotype_descriptio
n"),
                             mart=ensembl)

# select phenotypes related to pectoral fin
phenotypes <- grep("pectoral fin", unique(genes.to.phenotypes$phenotype_descrip
tion), value=T)

# select the genes annotated to select phenotypes
genes <- unique(genes.to.phenotypes$ensembl_gene_id[genes.to.phenotypes$phenoty
pe_description
                                                    %in% phenotypes])

# prepare the gene list vector
gene.list <- factor(as.integer(unique(genes.to.phenotypes$ensembl_gene_id) %in%
genes))
names(gene.list) <- unique(genes.to.phenotypes$ensembl_gene_id)
summary(gene.list)
```

```
##    0    1
## 2858  147
```

```r
# prepare the topAnat object based on topGO
top.anat.object <- topAnat(top.anat.data, gene.list)
```

```
##
## Checking topAnatData object.............
##
## Checking gene list......................
##
## Building most specific Ontology terms...  (  1173  Ontology terms found. )
##
## Building DAG topology...................  (  2035  Ontology terms and  3882
relations. )
##
## Annotating nodes (Can be long)..........  (  3005  genes annotated to the On
tology terms. )
```

```r
top.anat.object
```

```
##
## ------------------------- topGOdata object -------------------------
##
##  Description:
##    -  topAnatData class object, ready for anatomical ontology enrichment tes
t
##
##  Ontology:
##    -  UBERON ontology describing animal anatomical structures
##
##  3005 available genes (all genes from the array):
##    - symbol:  ENSDARG00000063924 ENSDARG00000028663 ENSDARG00000088634 ENSDA
RG00000058996 ENSDARG00000098696  ...
##    - 147  significant genes.
##
##  3005 feasible genes (genes that can be used in the analysis):
##    - symbol:  ENSDARG00000063924 ENSDARG00000028663 ENSDARG00000088634 ENSDA
RG00000058996 ENSDARG00000098696  ...
##    - 147  significant genes.
##
##  GO graph (nodes with at least  10  genes):
##    - a graph with directed edges
##    - number of nodes = 1081
##    - number of edges = 2032
##
## ------------------------- topGOdata object -------------------------
```

```
results <- runTest(top.anat.object, algorithm='weight', statistic='fisher')
```

```
##
##           -- Weight Algorithm --
##
##       The algorithm is scoring 1005 nontrivial nodes
##       parameters:
##           test statistic: fisher : ratio
```

```
##
##   Level 27:  1 nodes to be scored.
```

```
##
##   Level 26:  1 nodes to be scored.
```

```
##
##   Level 25:  1 nodes to be scored.
```

```
##
##   Level 24:  4 nodes to be scored.
```

```
##
##   Level 23:  4 nodes to be scored.
```

```
##
##   Level 22:  5 nodes to be scored.
```

```
##
##   Level 21:  4 nodes to be scored.
```

```
##
##   Level 20:  8 nodes to be scored.
```

```
##
##   Level 19:  23 nodes to be scored.
```

```
##
##   Level 18:  23 nodes to be scored.
```

```
##
##   Level 17:  27 nodes to be scored.
```

```
##
##   Level 16:  39 nodes to be scored.
```

```
##
##   Level 15:  63 nodes to be scored.
```

```
##
##   Level 14:  63 nodes to be scored.
```

```
##
##   Level 13:  74 nodes to be scored.
```

```
##
##   Level 12:  95 nodes to be scored.
```

```
##
##   Level 11:  119 nodes to be scored.
```

```
##
##   Level 10:  115 nodes to be scored.
```

```
##
##   Level 9:   92 nodes to be scored.
```

```
##
##   Level 8:   75 nodes to be scored.
```

```
##
##   Level 7:   67 nodes to be scored.
```

```
##
##   Level 6:   43 nodes to be scored.
```

```
##
##   Level 5:   27 nodes to be scored.
```

```
##
##   Level 4:   21 nodes to be scored.
```

```
##
##   Level 3:   6 nodes to be scored.
```

```
##
##   Level 2:   4 nodes to be scored.
```

```
##
##   Level 1:   1 nodes to be scored.
```

```
results
```

```
##
## Description: topAnatData class object, ready for anatomical ontology enrichm
ent test
## Ontology: UBERON ontology describing animal anatomical structures
## 'weight' algorithm with the 'fisher : ratio' test
## 1081 GO terms scored: 60 terms with p < 0.01
## Annotation data:
##     Annotated genes: 3005
##     Significant genes: 147
##     Min. no. of genes annotated to a GO: 10
##     Nontrivial nodes: 1005
```

```
# retrieve anatomical structures enriched at a 1% FDR threshold, sorted by decr
easing fold enrichment
table.over <- makeTable(top.anat.data, top.anat.object, results, cutoff=0.01)
```

```
##
## Building the results table for the 27 significant terms at FDR threshold of
0.01...
## Ordering results by pValue column in increasing order...
## Done
```

```
head(table.over)
```

```
##                        organId               organName annotated
## UBERON:0000151 UBERON:0000151             pectoral fin       439
## UBERON:0004357 UBERON:0004357      paired limb/fin bud       198
## UBERON:2000040 UBERON:2000040          median fin fold        59
## UBERON:0003051 UBERON:0003051              ear vesicle       391
## UBERON:0005729 UBERON:0005729 pectoral appendage field        20
## UBERON:0004376 UBERON:0004376                 fin bone        34
##                significant expected foldEnrichment       pValue
## UBERON:0000151          79    21.48       3.677840 1.358300e-27
## UBERON:0004357          48     9.69       4.953560 5.187251e-23
## UBERON:2000040          20     2.89       6.920415 9.370662e-13
## UBERON:0003051          49    19.13       2.561422 5.501734e-11
## UBERON:0005729          11     0.98      11.224490 3.052286e-10
## UBERON:0004376          12     1.66       7.228916 2.603199e-08
##                         FDR
## UBERON:0000151 1.468322e-24
## UBERON:0004357 2.803709e-20
## UBERON:2000040 3.376562e-10
## UBERON:0003051 1.486844e-08
## UBERON:0005729 6.599043e-08
## UBERON:0004376 4.690096e-06
```

```
# In order to retrieve significant genes mapped to the term " paired limb/fin b
ud"
term <- "UBERON:0004357"
termStat(top.anat.object, term)
```

```
##                Annotated Significant Expected
## UBERON:0004357       198          48     9.69
```

```
# 198 genes mapped to this term for Bgee 14.0 and Ensembl 84
genesInTerm(top.anat.object, term)
```

```
## $`UBERON:0004357`
##   [1] "ENSDARG00000001057" "ENSDARG00000001785" "ENSDARG00000002445"
##   [4] "ENSDARG00000002795" "ENSDARG00000002933" "ENSDARG00000002952"
##   [7] "ENSDARG00000003293" "ENSDARG00000003399" "ENSDARG00000004954"
##  [10] "ENSDARG00000005479" "ENSDARG00000005645" "ENSDARG00000005762"
##  [13] "ENSDARG00000006120" "ENSDARG00000006514" "ENSDARG00000007407"
##  [16] "ENSDARG00000007438" "ENSDARG00000007641" "ENSDARG00000008305"
##  [19] "ENSDARG00000008886" "ENSDARG00000009534" "ENSDARG00000011027"
##  [22] "ENSDARG00000011407" "ENSDARG00000011618" "ENSDARG00000012078"
##  [25] "ENSDARG00000012422" "ENSDARG00000012824" "ENSDARG00000013409"
##  [28] "ENSDARG00000013853" "ENSDARG00000013881" "ENSDARG00000014091"
##  [31] "ENSDARG00000014259" "ENSDARG00000014329" "ENSDARG00000014626"
##  [34] "ENSDARG00000014634" "ENSDARG00000014796" "ENSDARG00000015554"
##  [37] "ENSDARG00000015674" "ENSDARG00000016022" "ENSDARG00000016454"
##  [40] "ENSDARG00000016858" "ENSDARG00000017219" "ENSDARG00000018025"
##  [43] "ENSDARG00000018426" "ENSDARG00000018460" "ENSDARG00000018492"
##  [46] "ENSDARG00000018693" "ENSDARG00000018902" "ENSDARG00000019260"
##  [49] "ENSDARG00000019353" "ENSDARG00000019579" "ENSDARG00000019838"
##  [52] "ENSDARG00000019995" "ENSDARG00000020527" "ENSDARG00000021389"
##  [55] "ENSDARG00000021442" "ENSDARG00000021938" "ENSDARG00000022280"
##  [58] "ENSDARG00000024561" "ENSDARG00000024894" "ENSDARG00000025081"
##  [61] "ENSDARG00000025147" "ENSDARG00000025375" "ENSDARG00000025641"
##  [64] "ENSDARG00000025891" "ENSDARG00000028071" "ENSDARG00000029764"
##  [67] "ENSDARG00000030110" "ENSDARG00000030756" "ENSDARG00000030932"
##  [70] "ENSDARG00000031222" "ENSDARG00000031809" "ENSDARG00000031894"
##  [73] "ENSDARG00000031952" "ENSDARG00000033327" "ENSDARG00000033616"
##  [76] "ENSDARG00000034375" "ENSDARG00000035559" "ENSDARG00000035648"
##  [79] "ENSDARG00000036254" "ENSDARG00000036558" "ENSDARG00000037109"
##  [82] "ENSDARG00000037556" "ENSDARG00000037675" "ENSDARG00000037677"
##  [85] "ENSDARG00000038006" "ENSDARG00000038428" "ENSDARG00000038672"
##  [88] "ENSDARG00000038879" "ENSDARG00000038990" "ENSDARG00000038991"
##  [91] "ENSDARG00000040534" "ENSDARG00000040764" "ENSDARG00000041430"
##  [94] "ENSDARG00000041609" "ENSDARG00000041706" "ENSDARG00000041799"
##  [97] "ENSDARG00000042233" "ENSDARG00000042296" "ENSDARG00000043130"
## [100] "ENSDARG00000043559" "ENSDARG00000043923" "ENSDARG00000044511"
## [103] "ENSDARG00000044574" "ENSDARG00000052131" "ENSDARG00000052139"
## [106] "ENSDARG00000052344" "ENSDARG00000052494" "ENSDARG00000052652"
## [109] "ENSDARG00000053479" "ENSDARG00000053493" "ENSDARG00000054026"
## [112] "ENSDARG00000054030" "ENSDARG00000054619" "ENSDARG00000055026"
## [115] "ENSDARG00000055027" "ENSDARG00000055381" "ENSDARG00000055398"
## [118] "ENSDARG00000056995" "ENSDARG00000057830" "ENSDARG00000058115"
## [121] "ENSDARG00000058543" "ENSDARG00000058822" "ENSDARG00000059073"
## [124] "ENSDARG00000059233" "ENSDARG00000059276" "ENSDARG00000059279"
## [127] "ENSDARG00000059437" "ENSDARG00000060397" "ENSDARG00000060808"
## [130] "ENSDARG00000061328" "ENSDARG00000061345" "ENSDARG00000061600"
## [133] "ENSDARG00000062824" "ENSDARG00000068365" "ENSDARG00000068567"
## [136] "ENSDARG00000068732" "ENSDARG00000069105" "ENSDARG00000069473"
## [139] "ENSDARG00000069763" "ENSDARG00000069922" "ENSDARG00000070069"
## [142] "ENSDARG00000070670" "ENSDARG00000071336" "ENSDARG00000071560"
## [145] "ENSDARG00000071699" "ENSDARG00000073814" "ENSDARG00000074378"
## [148] "ENSDARG00000074597" "ENSDARG00000075713" "ENSDARG00000076010"
## [151] "ENSDARG00000076554" "ENSDARG00000076566" "ENSDARG00000076856"
```

```
## [154] "ENSDARG00000077121" "ENSDARG00000077353" "ENSDARG00000077473"
## [157] "ENSDARG00000078696" "ENSDARG00000078784" "ENSDARG00000078864"
## [160] "ENSDARG00000079027" "ENSDARG00000079570" "ENSDARG00000079922"
## [163] "ENSDARG00000079964" "ENSDARG00000080453" "ENSDARG00000087196"
## [166] "ENSDARG00000089805" "ENSDARG00000090820" "ENSDARG00000091161"
## [169] "ENSDARG00000092136" "ENSDARG00000092809" "ENSDARG00000095743"
## [172] "ENSDARG00000095859" "ENSDARG00000098359" "ENSDARG00000099088"
## [175] "ENSDARG00000099175" "ENSDARG00000099458" "ENSDARG00000099996"
## [178] "ENSDARG00000100236" "ENSDARG00000100312" "ENSDARG00000100725"
## [181] "ENSDARG00000101076" "ENSDARG00000101199" "ENSDARG00000101209"
## [184] "ENSDARG00000101244" "ENSDARG00000101701" "ENSDARG00000101766"
## [187] "ENSDARG00000101831" "ENSDARG00000102470" "ENSDARG00000102750"
## [190] "ENSDARG00000102824" "ENSDARG00000102995" "ENSDARG00000103432"
## [193] "ENSDARG00000103515" "ENSDARG00000103754" "ENSDARG00000104353"
## [196] "ENSDARG00000104815" "ENSDARG00000105230" "ENSDARG00000105357"
```

```
# 48 significant genes mapped to this term for Bgee 14.0 and Ensembl 84
annotated <- genesInTerm(top.anat.object, term)[["UBERON:0004357"]]
annotated[annotated %in% sigGenes(top.anat.object)]
```

```
##  [1] "ENSDARG00000002445" "ENSDARG00000002952" "ENSDARG00000003293"
##  [4] "ENSDARG00000008305" "ENSDARG00000011407" "ENSDARG00000012824"
##  [7] "ENSDARG00000013853" "ENSDARG00000013881" "ENSDARG00000014091"
## [10] "ENSDARG00000018426" "ENSDARG00000018693" "ENSDARG00000018902"
## [13] "ENSDARG00000019260" "ENSDARG00000019353" "ENSDARG00000019838"
## [16] "ENSDARG00000021389" "ENSDARG00000024894" "ENSDARG00000028071"
## [19] "ENSDARG00000030932" "ENSDARG00000031894" "ENSDARG00000036254"
## [22] "ENSDARG00000037677" "ENSDARG00000038006" "ENSDARG00000038672"
## [25] "ENSDARG00000041799" "ENSDARG00000042233" "ENSDARG00000042296"
## [28] "ENSDARG00000043559" "ENSDARG00000043923" "ENSDARG00000053493"
## [31] "ENSDARG00000054619" "ENSDARG00000058543" "ENSDARG00000060397"
## [34] "ENSDARG00000068567" "ENSDARG00000069473" "ENSDARG00000071336"
## [37] "ENSDARG00000073814" "ENSDARG00000076856" "ENSDARG00000077121"
## [40] "ENSDARG00000077353" "ENSDARG00000079027" "ENSDARG00000079570"
## [43] "ENSDARG00000087196" "ENSDARG00000095859" "ENSDARG00000099088"
## [46] "ENSDARG00000100312" "ENSDARG00000101831" "ENSDARG00000105357"
```

```
sessionInfo()
```

```
## R version 3.5.0 (2018-04-23)
## Platform: x86_64-apple-darwin15.6.0 (64-bit)
## Running under: macOS Sierra 10.12.1
##
## Matrix products: default
## BLAS: /Library/Frameworks/R.framework/Versions/3.5/Resources/lib/libRblas.0.
dylib
## LAPACK: /Library/Frameworks/R.framework/Versions/3.5/Resources/lib/libRlapac
k.dylib
##
## locale:
## [1] en_US.UTF-8/en_US.UTF-8/en_US.UTF-8/C/en_US.UTF-8/en_US.UTF-8
##
## attached base packages:
##  [1] tcltk     stats4    parallel  stats     graphics  grDevices utils
##  [8] datasets  methods   base
##
## other attached packages:
##  [1] biomaRt_2.36.1       edgeR_3.22.2         limma_3.36.1
##  [4] Mfuzz_2.40.0         DynDoc_1.58.0        widgetTools_1.58.0
##  [7] e1071_1.6-8          BgeeDB_2.6.2         tidyr_0.8.1
## [10] topGO_2.32.0         SparseM_1.77         GO.db_3.6.0
## [13] AnnotationDbi_1.42.1 IRanges_2.14.10      S4Vectors_0.18.3
## [16] Biobase_2.40.0       graph_1.58.0         BiocGenerics_0.26.0
## [19] BiocInstaller_1.30.0
##
## loaded via a namespace (and not attached):
##  [1] Rcpp_0.12.17       locfit_1.5-9.1     lattice_0.20-35
##  [4] prettyunits_1.0.2  class_7.3-14       assertthat_0.2.0
##  [7] rprojroot_1.3-2    digest_0.6.15      R6_2.2.2
## [10] backports_1.1.2    RSQLite_2.1.1      evaluate_0.10.1
## [13] httr_1.3.1         pillar_1.2.3       rlang_0.2.1
## [16] progress_1.2.0     curl_3.2           data.table_1.11.4
## [19] blob_1.1.1         rmarkdown_1.10     stringr_1.3.1
## [22] RCurl_1.95-4.10    bit_1.1-14         compiler_3.5.0
## [25] pkgconfig_2.0.1    tkWidgets_1.58.0   htmltools_0.3.6
## [28] tidyselect_0.2.4   tibble_1.4.2       matrixStats_0.53.1
## [31] XML_3.98-1.11      crayon_1.3.4       dplyr_0.7.5
## [34] bitops_1.0-6       grid_3.5.0         DBI_1.0.0
## [37] magrittr_1.5       stringi_1.2.3      bindrcpp_0.2.2
## [40] tools_3.5.0        bit64_0.9-7        glue_1.2.0
## [43] purrr_0.2.5        hms_0.4.2          yaml_2.1.19
## [46] memoise_1.1.0      knitr_1.20         bindr_0.1.1
```
