# Supplementary material for: BgeeDB, an R package for retrieval of curated expression datasets and for gene list expression localization enrichment tests
Source: F1000Res. 2018 Aug 7;5:2748. Originally published 2016 Nov 23. [Version 2] doi: 10.12688/f1000research.9973.2 (PMC6113886; doi:10.12688/f1000research.9973.2)
Supplement: Peer review history

# Peer review: Referee response for version 2

**DOI:** 10.5256/f1000research.16883.r36881

**Published:** 2018-08-28

**Authors:** Leonardo Collado-Torres

**Affiliations:** 1 Lieber Institute for Brain Development, Baltimore, MD, USA

The authors (including the new author J. Wollbrett) have addressed all my comments from version 1 very throughly or plan to address some of the more technical ones in the future. 

 I want to highlight all the work the authors did ensuring that their paper is reproducible (they mention versions of packages throughout the text) and in clarifying their work (mostly updates on the introduction). I was able to download supplementary file 1 (an .Rmd file) and execute it without any problems or modifications at all using R 3.5.1 with Bioconductor 3.7 on a Mac (BgeeDB 2.6.2, MFuzz 2.40.0, biomaRt 2.36.1). The authors did a great job with https://github.com/BgeeDB/bgee_pipeline which I believe includes all the information needed for anyone who is interested in the Bgee pipeline. If not, the authors seem responsive on https://github.com/BgeeDB/bgee_pipeline/issues and https://github.com/BgeeDB/BgeeDB_R/issues which is a great sign. Thanks to their changes in the introduction I now have a better understanding of their anatomical expression enrichment test. 

 I look forward to seeing how the community uses BgeeDB in the future and the use cases they apply it to.

I have read this submission. I believe that I have an appropriate level of expertise to confirm that it is of an acceptable scientific standard.

# Peer review: Referee response for version 1

**DOI:** 10.5256/f1000research.10748.r17980

**Published:** 2016-12-16

**Authors:** Leonardo Collado-Torres

**Affiliations:** 1 Lieber Institute for Brain Development, Baltimore, MD, USA

In this manuscript the authors describe the BgeeDB Bioconductor package and show how to use it (as of Bioconductor 3.4) to interact with Bgee ^1^ in order to get the data from Bgee into your R session. This allows users to then perform differential expression analyses and integrate Bgee with other data sets such as unpublished data. The manuscript includes code that shows how to use BgeeDB and showcases it's different features including their unique anatomical expression enrichment analysis method.

 I find interesting that you can use BgeeDB to get data from different platforms and from different organisms. Most of this can be done using other packages such as GEOquery, but BgeeDB makes it so the user doesn't have to do all the processing of the data and standarization over multiple projects.

 My main concern with the manuscript in its current form and the BgeeDB package itself is the lack of clarity on how the data has been processed and how the anatomical expression test works. That is, it could potentially become a black box that produces interesting output but hides information that could be important.

 For example, I'm sure some of the Affymetrix data could be downloaded with other packages and I do not know what would be the differences between the raw data and the data downloaded via BgeeDB. Is the data in BgeeDB normalized? If so, how? The help pages of BgeeDB, the package vignette, the original Bgee publication ^1^ and http://bgee.org/?page=doc did not help me fully answer these questions (I might have missed the information). Maybe the functions in BgeeDB could print a message describing the main steps of how a given data set was processed or this could be added to the help pages. I currently ignore if all data sets were treated the same. For instance, is all the Affymetrix data normalized with the same method and same parameters? I assume that the answer is yes but I don't know. I suggest that the authors describe in more detail the data available in Bgee. The authors might want to consider making the processing code public at https://github.com/BgeeDB or citable via figshare.

 With the anatomical expression test it's not clear to me how to interpret the results from BgeeDB::makeTable(). I understand that the authors will describe the details of how their anatomical test works in a future publication, which they did before with Bgee ^1^ and Homolanto ^2^. Ideally, the anatomical expression test would have been described first followed by BgeeDB. Without hindering the current plan, I believe that the authors could provide a summary of how TopAnat works. Then they can explain it fully in the planned future TopAnat publication. I am also curious on how users could use their own data to improve the TopAnat results, although that could be work for the TopAnat paper or future work.

 I think that the manuscript is overall well written and will be more appealing if the data and main features (TopAnat) are described in more detail. I hope that the authors are not discouraged by my report. 

 Best,

 Leonardo

 Minor comments:

- I'm an author of recount ^3^ which is incorrectly cited here. The pre-print version of https://jhubiostatistics.shinyapps.io/recount/ had data from 2040 different projects which together made up more than 60,000 RNA-seq samples. The current version has data from over 70,000 Illumina human RNA-seq samples from SRA, GTEx and TCGA.
- I don't think that it makes sense to include the str() calls in the paper. They do make sense in the supplementary material (the html and pdf rendered versions of the paper code) since those include the output. Also, while str() shows all the details of an object, it can encourage users to write code that depends on the internal structure of the object. You might want to consider adding accessor functions.
- If you added indentation the code that runs over multiple lines would be easier to read. You can use the Bioconductor standard of using 4 spaces at the start of the line. Also make sure that object names don't get split into multiple lines. For example check the line after the "list experiments including both brain and liver samples" comment where "Anatomical.entity.ID" gets split into "Anatomical.e" and "ntity.ID" in the html version of the paper. Copy pasting works fine, but if someone prints the paper they might introduce errors can be avoided with better formatting. F1000Research's team should be able to tell you what is the character limit per line to use so that the PDF and HTML versions look great. The formatR package might be useful here.
- I would not use numerical indexes in the code since the results could change with time in such a way that the current code would not work in the future or worse, it might run without error but change the results in a way a new user would not notice. For example, change the code on the line after the "order developmental stages" comment which currently reads:
  data.E.MEXP.51.formatted <- data.E.MEXP.51.formatted[, c(5,8,9,3,2,1,4,7,6)]
- The comment that reads with "retrieve anatomical structures enriched at a 1% FDR threshold" is mixed with the code. That is, you are missing a new line character.
- Reference 46 is incorrect. It's edgeR, not eedgeR.
- The package's vignette is missing a title as currently shown at http://bioconductor.org/packages/release/bioc/html/BgeeDB.html.
- I recommend adding internal R links to your manual pages. For example, ?topAnat mentions loadTopAnatData(). Those links make it easier for a user to browse the help pages.

I was able to run all the code without any edits (beyond that new line issue I already mentioned) using Bioconductor 3.4 (current Bioc-release) on R 3.3.1. Here are my session details:

 > options(width = 120)

 > devtools::session_info()

 Session info -----------------------------------------------------------------------------------------------------------

  setting  value                       

  version  R version 3.3.1 (2016-06-21)

  system   x86_64, mingw32             

  ui       RStudio (0.99.902)          

  language (EN)                        

  collate  English_United States.1252  

  tz       America/Mexico_City         

  date     2016-12-15                  

 Packages ---------------------------------------------------------------------------------------------------------------

  package       * version  date       source        

  AnnotationDbi * 1.36.0   2016-10-18 Bioconductor  

  assertthat      0.1      2013-12-06 CRAN (R 3.3.1)

  BgeeDB        * 2.0.0    2016-10-18 Bioconductor  

  Biobase       * 2.34.0   2016-10-18 Bioconductor  

  BiocGenerics  * 0.20.0   2016-10-18 Bioconductor  

  BiocInstaller * 1.24.0   2016-10-18 Bioconductor  

  biomaRt       * 2.30.0   2016-10-18 Bioconductor  

  bitops          1.0-6    2013-08-17 CRAN (R 3.3.1)

  class           7.3-14   2015-08-30 CRAN (R 3.3.1)

  data.table      1.10.0   2016-12-03 CRAN (R 3.3.2)

  DBI             0.5-1    2016-09-10 CRAN (R 3.3.1)

  devtools        1.12.0   2016-06-24 CRAN (R 3.3.1)

  digest          0.6.10   2016-08-02 CRAN (R 3.3.1)

  dplyr           0.5.0    2016-06-24 CRAN (R 3.3.1)

  DynDoc        * 1.52.0   2016-10-18 Bioconductor  

  e1071         * 1.6-7    2015-08-05 CRAN (R 3.3.1)

  edgeR         * 3.16.4   2016-11-27 Bioconductor  

  GO.db         * 3.4.0    2016-10-22 Bioconductor  

  graph         * 1.52.0   2016-10-18 Bioconductor  

  IRanges       * 2.8.1    2016-11-08 Bioconductor  

  lattice         0.20-34  2016-09-06 CRAN (R 3.3.1)

  limma         * 3.30.6   2016-11-29 Bioconductor  

  locfit          1.5-9.1  2013-04-20 CRAN (R 3.3.1)

  magrittr        1.5      2014-11-22 CRAN (R 3.3.1)

  matrixStats     0.51.0   2016-10-09 CRAN (R 3.3.1)

  memoise         1.0.0    2016-01-29 CRAN (R 3.3.1)

  Mfuzz         * 2.34.0   2016-10-18 Bioconductor  

  R6              2.2.0    2016-10-05 CRAN (R 3.3.1)

  Rcpp            0.12.8   2016-11-17 CRAN (R 3.3.2)

  RCurl           1.95-4.8 2016-03-01 CRAN (R 3.3.1)

  rsconnect       0.6      2016-11-21 CRAN (R 3.3.2)

  RSQLite         1.1-1    2016-12-10 CRAN (R 3.3.2)

  S4Vectors     * 0.12.1   2016-12-01 Bioconductor  

  SparseM       * 1.74     2016-11-10 CRAN (R 3.3.2)

  tibble          1.2      2016-08-26 CRAN (R 3.3.1)

  tidyr         * 0.6.0    2016-08-12 CRAN (R 3.3.1)

  tkWidgets       1.52.0   2016-10-18 Bioconductor  

  topGO         * 2.26.0   2016-10-18 Bioconductor  

  widgetTools   * 1.52.0   2016-10-18 Bioconductor  

  withr           1.0.2    2016-06-20 CRAN (R 3.3.1)

  XML             3.98-1.5 2016-11-10 CRAN (R 3.3.2)

 Regarding Virag Sharma's peer review report ^4^, I assume that Virag was using an earlier R version (and thus an earlier Bioconductor version). The current development version of BgeeDB uses "dataType" and not "datatype", just like the release version. Check https://github.com/Bioconductor-mirror/BgeeDB/search?utf8=%E2%9C%93&q=datatype. Hopefully the authors won't change the spelling of arguments in the future since that's confusing for users, although that's certainly doable following the deprecated/defunct code cycle.

 Regarding Daniel S. Himmelstein's peer review report ^5^, there is no need to add a license file when the license is specified in the DESCRIPTION file of an R package. See https://github.com/Bioconductor-mirror/BgeeDB/blob/master/DESCRIPTION#L14 where they state that the license is GPL-2. Although the authors should make sure that they correctly specify which license their software is released on: GPL-2 or GPLv3 as Daniel mentioned. Regarding where to place bug reports, the authors could resolve this by specifying the "BugReports" field in their DESCRIPTION file. For example see https://github.com/Bioconductor-mirror/recount/blob/master/DESCRIPTION#L63. I also agree with Daniel that currently BgeeDB has a bit of a messy download structure. I would prefer if the files were downloaded in a single directory (say "bgee_downloads") instead of the current working directory.

I have read this submission. I believe that I have an appropriate level of expertise to state that I do not consider it to be of an acceptable scientific standard, for reasons outlined above.

## References

[1] BastianFParmentierGRouxJMorettiSLaudetVRobinson-RechaviM: Bgee: Integrating and Comparing Heterogeneous Transcriptome Data Among Species.2008; 10.1007/978-3-540-69828-9_12 124-131 10.1007/978-3-540-69828-9_12

[2] ParmentierGBastianFBRobinson-RechaviM: Homolonto: generating homology relationships by pairwise alignment of ontologies and application to vertebrate anatomy. *Bioinformatics*.2010;26(14) : 10.1093/bioinformatics/btq283 1766-71 10.1093/bioinformatics/btq283 20519284PMC2894521

[3] Collado-TorresLNelloreAKammersKEllisSTaubMHansenKJaffeALangmeadBLeekJ: recount: A large-scale resource of analysis-ready RNA-seq expression data.2016; 10.1101/068478 10.1101/068478

[4] SharmaV: Referee Report For: BgeeDB, an R package for retrieval of curated expression datasets and for gene list expression localization enrichment tests [version 1; referees: 1 approved, 1 approved with reservations]. *F1000Research*.2016;5(2748) : 10.5256/f1000research.10748.r17925 10.5256/f1000research.10748.r17925 PMC611388630467516

[5] HimmelsteinDS: Referee Report For: BgeeDB, an R package for retrieval of curated expression datasets and for gene list expression localization enrichment tests [version 1; referees: 1 approved, 1 approved with reservations].2016;5(2748) : 10.5256/f1000research.10748.r18221 10.5256/f1000research.10748.r18221 PMC611388630467516

# Response

**Published:** 2018-06-28

**Authors:** Frederic Bastian

**Affiliations:** Swiss Institute of Bioinformatics - University of Lausanne, Switzerland

*My main concern with the manuscript in its current form and the BgeeDB package itself is the lack of clarity on how the data has been processed and how the anatomical expression test works. That is, it could potentially become a black box that produces interesting output but hides information that could be important.*

*For example, I'm sure some of the Affymetrix data could be downloaded with other packages and I do not know what would be the differences between the raw data and the data downloaded via BgeeDB. Is the data in BgeeDB normalized? If so, how? The help pages of BgeeDB, the package vignette, the original Bgee publication1 and http://bgee.org/?page=doc did not help me fully answer these questions (I might have missed the information).*

 We have made public the Bgee pipeline source code at https://github.com/BgeeDB/bgee_pipeline. We also have added a paragraph at the end of the "Introduction" section, pointing to the relevant part of the documentation for RNA-Seq and Affymetrix analyses, and describing them in brief.

 ---

*Maybe the functions in BgeeDB could print a message describing the main steps of how a given data set was processed or this could be added to the help pages.*

 We have opened an issue on our tracker related to this point, see https://github.com/BgeeDB/BgeeDB_R/issues/22. We will add a function pointing to the relevant documentation in a future release.

 ---

*I currently ignore if all data sets were treated the same. For instance, is all the Affymetrix data normalized with the same method and same parameters? I assume that the answer is yes but I don't know.*

 The Affymetrix data are not treated in the same way depending on whether the raw data were available, or only the data processed by using the MAS5 software. This is clarified at the end of the "Introduction" section. Also, in the package, this information about raw data availability can be retrieved in the annotation data frame.

 ---

*I suggest that the authors describe in more detail the data available in Bgee. The authors might want to consider making the processing code public at https://github.com/BgeeDB or citable via figshare.*

 We have made public the Bgee pipeline source code at https://github.com/BgeeDB/bgee_pipeline.

 ---

*With the anatomical expression test it's not clear to me how to interpret the results from BgeeDB::makeTable(). I understand that the authors will describe the details of how their anatomical test works in a future publication, which they did before with Bgee and Homolanto. Ideally, the anatomical expression test would have been described first followed by BgeeDB. Without hindering the current plan, I believe that the authors could provide a summary of how TopAnat works. Then they can explain it fully in the planned future TopAnat publication.*

 We have added a brief description of how TopAnat works in the "Introduction" section.

 ---

*I am also curious on how users could use their own data to improve the TopAnat results, although that could be work for the TopAnat paper or future work.*

 This represents an advanced use of TopAnat that we don't find suitable for the paper. But users can override the association file, mapping genes to anatomical structures in the BgeeDB directory, to use their own data. Also, since the source code of the package is public, users can also modify the mapping files used by modifying the source code.

 ---

*I'm an author of recount which is incorrectly cited here. The pre-print version of https://jhubiostatistics.shinyapps.io/recount/ had data from 2040 different projects which together made up more than 60,000 RNA-seq samples. The current version has data from over 70,000 Illumina human RNA-seq samples from SRA, GTEx and TCGA.*

 We have updated the number in our paper. We apologize for the mistake.

 ---

*I don't think that it makes sense to include the str() calls in the paper. They do make sense in the supplementary material (the html and pdf rendered versions of the paper code) since those include the output. Also, while str() shows all the details of an object, it can encourage users to write code that depends on the internal structure of the object. You might want to consider adding accessor functions.*

 We have removed str() calls from the paper. For the future, we will think of adding accessor functions, although several are already available thanks to the topGO package.

 ---

*If you added indentation the code that runs over multiple lines would be easier to read. You can use the Bioconductor standard of using 4 spaces at the start of the line. Also make sure that object names don't get split into multiple lines. For example check the line after the "list experiments including both brain and liver samples" comment where "Anatomical.entity.ID" gets split into "Anatomical.e" and "ntity.ID" in the html version of the paper. Copy pasting works fine, but if someone prints the paper they might introduce errors can be avoided with better formatting. F1000Research's team should be able to tell you what is the character limit per line to use so that the PDF and HTML versions look great. The formatR package might be useful here.*

 We didn't know about the formatR package and will have a look at it. In the meantime, we have split such offending lines, as identified by the reviewer.

 ---

*I would not use numerical indexes in the code since the results could change with time in such a way that the current code would not work in the future or worse, it might run without error but change the results in a way a new user would not notice. For example, change the code on the line after the "order developmental stages" comment which currently reads:*

*data.E.MEXP.51.formatted <- data.E.MEXP.51.formatted[, c(5,8,9,3,2,1,4,7,6)]*

 We have replaced all lines using numerical indexes, with use of column names.

 ---

*The comment that reads with "retrieve anatomical structures enriched at a 1% FDR threshold" is mixed with the code. That is, you are missing a new line character.*

 This was fixed.

 ---

*Reference 46 is incorrect. It's edgeR, not eedgeR.*

 This was fixed.

 ---

*The package's vignette is missing a title as currently shown at http://bioconductor.org/packages/release/bioc/html/BgeeDB.html.*

 This was added.

 ---

*I recommend adding internal R links to your manual pages. For example, ?topAnat mentions loadTopAnatData(). Those links make it easier for a user to browse the help pages.*

 We thank the reviewer for the suggestion, and we will implement this in a future release.

 ---

*I was able to run all the code without any edits (beyond that new line issue I already mentioned) using Bioconductor 3.4 (current Bioc-release) on R 3.3.1. Here are my session details:*

*> options(width = 120)*

*> devtools::session_info()*

*[...]*

*Regarding Virag Sharma's peer review report4, I assume that Virag was using an earlier R version (and thus an earlier Bioconductor version). The current development version of BgeeDB uses "dataType" and not "datatype", just like the release version. Check https://github.com/Bioconductor-mirror/BgeeDB/search?utf8=%E2%9C%93&q=datatype. Hopefully the authors won't change the spelling of arguments in the future since that's confusing for users, although that's certainly doable following the deprecated/defunct code cycle.*

 This is indeed a change that we introduced in an earlier version, in an effort to name all our arguments in a consistent manner. We will try not to change this in the future.

 ---

*Regarding Daniel S. Himmelstein's peer review report5, there is no need to add a license file when the license is specified in the DESCRIPTION file of an R package. See https://github.com/Bioconductor-mirror/BgeeDB/blob/master/DESCRIPTION#L14 where they state that the license is GPL-2. Although the authors should make sure that they correctly specify which license their software is released on: GPL-2 or GPLv3 as Daniel mentioned.*

 We have updated the DESCRIPTION file in the development branch of Bioconductor. The package is now released under the GPL-3.0 license.

 ---

*Regarding where to place bug reports, the authors could resolve this by specifying the "BugReports" field in their DESCRIPTION file. For example see https://github.com/Bioconductor-mirror/recount/blob/master/DESCRIPTION#L63.*

 This was done.

 ---

*I also agree with Daniel that currently BgeeDB has a bit of a messy download structure. I would prefer if the files were downloaded in a single directory (say "bgee_downloads") instead of the current working directory.*

 While another directory can be specified by using the "pathToData" argument, it is true that the solution proposed by the reviewer would be convenient, and we will try to update the package accordingly in the future.

# Peer review: Referee response for version 1

**DOI:** 10.5256/f1000research.10748.r18221

**Published:** 2016-12-14

**Authors:** Daniel S. Himmelstein

**Affiliations:** 1 Systems Pharmacology and Translational Therapeutics, Perelman School of Medicine, University of Pennsylvania, Philadelphia, PA, USA

This study describes the BgeeDB R package, which provides a programmatic interface for accessing Bgee gene expression data. Bgee is a valuable resource because it integrates gene expression results across many experiments. Previously, I've used Bgee for its presence/absence of expression calls and its differential expression calls.

 In my opinion, Bgee's ability to provide a genome-wide profile of expression for a given species, developmental stage, and anatomical structure is its most powerful capability. It was not clear to me whether BgeeDB provides this functionality. For example, can the user retrieve the normalized expression level across several experiments for the same species-stage-anatomy combination? In general, I think users will be more interested in this high-level functionality than the low level access BgeeDB currently provides. An example here would likely clear things up for me.

 Is it possible to integrate expression levels across Affymetrix and RNA-Seq experiments?

 The Zenodo archive of the source code specifies GPLv3 as the license. This is great, but it's ideal to also add a LICENSE file to the GitHub.

 It looks like there are at least two potential places where bug reports should be filed: on Bioconductor Support and GitHub Issues. It would be nice to clarify the preferred location for filing bug reports go and opening pull requests.

 Currently, the GitHub repository BgeeDB/BgeeDB_R mentioned in the manuscript is forked from wirawara/BgeeDB. I expect this may cause some confusion, as BgeeDB/BgeeDB_R should be the upstream repository that users fork and contribute back to. If you make wirawara/BgeeDB private, this should break the relationship. @wirawara can then fork BgeeDB/BgeeDB_R to continue contributions if desired.

 Finally, I created some GitHub issues as part of this review:

- Sample annotation variable names
- A less messy default download directory

I have read this submission. I believe that I have an appropriate level of expertise to confirm that it is of an acceptable scientific standard, however I have significant reservations, as outlined above.

## References

[1] MorinAUrbanJSlizP: A quick guide to software licensing for the scientist-programmer. *PLoS Comput Biol*.2012;8(7) : 10.1371/journal.pcbi.1002598 e1002598 10.1371/journal.pcbi.1002598 22844236PMC3406002

# Response

**Published:** 2018-06-28

**Authors:** Frederic Bastian

**Affiliations:** Swiss Institute of Bioinformatics - University of Lausanne, Switzerland

*In my opinion, Bgee's ability to provide a genome-wide profile of expression for a given species, developmental stage, and anatomical structure is its most powerful capability. It was not clear to me whether BgeeDB provides this functionality. For example, can the user retrieve the normalized expression level across several experiments for the same species-stage-anatomy combination? In general, I think users will be more interested in this high-level functionality than the low level access BgeeDB currently provides. An example here would likely clear things up for me.*

 Indeed there is currently no easy way to do this. As mentioned in https://github.com/BgeeDB/BgeeDB_R/issues/7, it would be nice to have a getDataByCondition function that would return all processed data for chips / libraries matching a queried organ/stage/(sex)/(strain). But it was hard to set priorities for the initial development (should the package complement the web interface, or be orthogonal to it?), and we will likely implement it in the near future.

 ---

*Is it possible to integrate expression levels across Affymetrix and RNA-Seq experiments?*

 If the reviewer means to integrate present/absent expression calls, it is relatively easy to get all the genes expressed in one tissue and all sub-tissues from Affymetrix and RNA-Seq data, although a dedicated method could be added, for instance:

 library(BgeeDB)

 bgee_human <- Bgee$new(species='Homo_sapiens', dataType=c('rna_seq', 'affymetrix'))

 my_data <- loadTopAnatData(bgee_human)

 calls_by_tissue <- reverseSplit(my_data$gene2anatomy)

 # pick you favorite tissue, for example liver

 calls_by_tissue[["UBERON:0002107"]]

 And this can be limited by stage too, for example:

 my_data <- loadTopAnatData(bgee_human, stage="UBERON:0000068")

 We have noted in the issue 7 mentioned above to add a direct function to do this.

 If the reviewer means to integrate levels of expression, it is then not the aim of Bgee: Bgee integrate different data types and different experiments, processed and normalized independently (but in a consistent manner).

 ---

*The Zenodo archive of the source code specifies GPLv3 as the license. This is great, but it's ideal to also add a LICENSE file to the GitHub.*

 We have added the LICENSE file to GitHub (GPL 3.0).

 ---

*It looks like there are at least two potential places where bug reports should be filed: on Bioconductor Support and GitHub Issues. It would be nice to clarify the preferred location for filing bug reports go and opening pull requests.*

 We have added the preferred location for filing bug reports at the end of the "Introduction" section (GitHub), and in the DESCRIPTION file of the source code.

 ---

*Currently, the GitHub repository BgeeDB/BgeeDB_R mentioned in the manuscript is forked from wirawara/BgeeDB. I expect this may cause some confusion, as BgeeDB/BgeeDB_R should be the upstream repository that users fork and contribute back to. If you make wirawara/BgeeDB private, this should break the relationship. @wirawara can then fork BgeeDB/BgeeDB_R to continue contributions if desired.*

 We thank the reviewer for the suggestion, we have now made wirawara/BgeeDB private.

 ---

*Finally, I created some GitHub issues as part of this review:*

*Sample annotation variable names*

*https://github.com/BgeeDB/BgeeDB_R/issues/5*

 We have replied on the issue. Our answer was that is a bit of a controversial topic. For example Google's R Style Guide (https://google.github.io/styleguide/Rguide.xml#identifiers) advise against the use of underscores (although they do not justify why, and we agree that the "words separated with dots" convention can be disturbing for python users).

 ---

*A less messy default download directory*

 This point was discussed in https://github.com/BgeeDB/BgeeDB_R/issues/4. We notably mention that another directory can be specified by using the "pathToData" argument. This parameter is mentioned at the end of the section "Data download and import of normalized expression levels". We agree that a default directory should be used in future releases.

# Peer review: Referee response for version 1

**DOI:** 10.5256/f1000research.10748.r17925

**Published:** 2016-12-07

**Authors:** Virag Sharma

**Affiliations:** 1 Max Planck Institute of Molecular Cell Biology and Genetics (MPI-CBG), Dresden, Germany; 2 Max Planck Institute for the Physics of Complex Systems, Dresden, Germany

In the manuscript, Komljenovic et al. present BgeeDB which is an R package for retrieval of expression datasets which have been curated. Additionally, they also provide a method (TopAnat) to determine tissue-specific enrichments for a given list of genes and species.

 The former is a very useful resource because there is clearly a need for a database that provides gene expression datasets which are homogenous in nature and are of comparable quality. The BgeeDB database contains gene expression datasets from 17 species across different tissues and developmental stages, which is impressive. The fact that the database can be queried via a Bioconductor package should ensure that the database will be used  by both - wet-lab biologists and computational scientists.

 Similarly, the TopAnat method also provides a useful functionality to determine anatomical expression enrichment on a user specified list.

 I have a few minor comments regarding the manuscript:

1. The authors should include some details about how they have reprocessed the gene expression datasets that are a part of BgeeDB. At the moment, it is rather unclear how this was achieved. I assume that the authors have an automated pipeline in place but it would be beneficial for readers to know how this was done.
2. The authors state that “TopAnat allows for discovery of tissues where a set of genes is preferentially expressed”. Is TopAnat the only tool that offers such a functionality? A brief background of similar tools that are currently available will be useful for the readers.
3. I was not able to run the workflow that the authors have included in the Supplementary material:
  See below: 
  source("https://bioconductor.org/biocLite.R")
  biocLite("BgeeDB")
  biocLite(c("edgeR", "Mfuzz", "biomaRt"))
  library(BgeeDB)
  listBgeeSpecies()
  bgee_affymetrix <- Bgee$new(species="Mus_musculus", dataType="affymetrix", release="13.2")
  Error in envRefSetField(.Object, field, classDef, selfEnv, elements[[field]]) :
  'dataType' is not a field in class "Bgee"
  ## Turns out that I need to use "datatype" instead of "dataType"
  bgee_affymetrix <- Bgee$new(species="Mus_musculus", datatype="affymetrix")  
  bgee_affymetrix <- Bgee$new(species="Mus_musculus", datatype="affymetrix", release="13.2")
  Error in envRefSetField(.Object, field, classDef, selfEnv, elements[[field]]) :
  'release' is not a field in class "Bgee"
  ####
  At this moment, I did not try further.
  The authors need to clearly state what version of BgeeDB was used to create this workflow. If something has changed, then this needs to be appropriately addressed. I tried using “release=13.2” but it did not work.
4. I did manage to run an enrichment test for anatomical terms though with some tweaking
  ## Again an error message
  bgee_topanat <- loadTopAnatData(species="Danio_rerio")
  Error in loadTopAnatData(species = "Danio_rerio") :
  Problem: the specified speciesId is not among the list of species in Bgee.
  ## This works though
  myTopAnatData <- loadTopAnatData(species="7955")
  ####
  The rest of the work-flow went smoothly and I was able to get a list of anatomical structures sorted by their p-value
  head(tableOver)
          organId                           organName annotated significant
  12 UBERON:0004357                 paired limb/fin bud 144          41
  2  UBERON:0000151                        pectoral fin 420          70
  22 UBERON:2000040                     median fin fold 51          18
  9  UBERON:0003051                         ear vesicle 304          41
  15 UBERON:0005729            pectoral appendage field 16          10
  16 UBERON:0007390 pectoral appendage cartilage tissue 17           9
   expected foldEnrichment       pValue          FDR
  12     7.15       5.734266 1.622480e-22 1.445630e-19
  2     20.85       3.357314 1.037552e-18 4.622296e-16
  22     2.53       7.114625 7.171001e-12 2.129787e-09
  9     15.09       2.717031 3.135769e-10 6.984926e-08
  15     0.79      12.658228 4.004917e-10 7.136762e-08
  16     0.84      10.714286 2.411891e-08 3.581659e-06
  It would be useful if the authors could include a feature that allows the TopAnat method to print the 41 genes which represent the paired limb/fin bud. At some point, the users might want to revisit their gene lists and tag their genes based on the different anatomical structures.
  Other tools that perform Enrichment tests, for example Enrichr ^1^, have this feature and this is extremely useful, in my opinion.

I have read this submission. I believe that I have an appropriate level of expertise to confirm that it is of an acceptable scientific standard.

## References

[1] KuleshovMVJonesMRRouillardADFernandezNFDuanQWangZKoplevSJenkinsSLJagodnikKMLachmannAMcDermottMGMonteiroCDGundersenGWMa'ayanA: Enrichr: a comprehensive gene set enrichment analysis web server 2016 update. *Nucleic Acids Res*.2016;44(W1) : 10.1093/nar/gkw377 W90-7 10.1093/nar/gkw377 27141961PMC4987924

# Response

**Published:** 2018-06-28

**Authors:** Frederic Bastian

**Affiliations:** Swiss Institute of Bioinformatics - University of Lausanne, Switzerland

*The authors should include some details about how they have reprocessed the gene expression datasets that are a part of BgeeDB. At the moment, it is rather unclear how this was achieved. I assume that the authors have an automated pipeline in place but it would be beneficial for readers to know how this was done.*

 There is now a complete and updated documentation for the Bgee pipeline: https://github.com/BgeeDB/bgee_pipeline

 We have included this information in the manuscript, as well as a brief outline of the analyses we perform, see "Introduction" section.

 ---

*The authors state that “TopAnat allows for discovery of tissues where a set of genes is preferentially expressed”. Is TopAnat the only tool that offers such a functionality? A brief background of similar tools that are currently available will be useful for the readers.*

 We have added a paragraph describing similar tools, see end of the "Introduction" section.

 ---

*I was not able to run the workflow that the authors have included in the Supplementary material:*

*[...]*

*At this moment, I did not try further.*

*The authors need to clearly state what version of BgeeDB was used to create this workflow. If something has changed, then this needs to be appropriately addressed. I tried using “release=13.2” but it did not work.*

 We suspect that the reviewer did not use the latest version of the package (maybe the Bioconductor release itself needs to be updated first). The reviewer could maybe uninstall the BgeeDB package and rerun the following steps:

 source("https://bioconductor.org/biocLite.R")

 biocLite("BgeeDB")

 sessionInfo()

 With a package version >= 2.6.2, the errors should disappear. The R, Bioconductor, and BgeeDB package version requirements are listed at the beginning of the "Methods" section.

 If the problem persists, could the reviewer post the sessionInfo() results?

 Of note, the “release” argument is used to specify a particular Bgee release, but this is independent of the package version.

 ---

*I did manage to run an enrichment test for anatomical terms though with some tweaking*

*## Again an error message*

*bgee_topanat <- loadTopAnatData(species="Danio_rerio")*

*Error in loadTopAnatData(species = "Danio_rerio") :*

*Problem: the specified speciesId is not among the list of species in Bgee.*

*## This works though*

*myTopAnatData <- loadTopAnatData(species="7955")*

*####*

 Again, this should be solved by updating to the last BgeeDB version

 ---

*The rest of the work-flow went smoothly and I was able to get a list of anatomical structures sorted by their p-value*

*[...]*

*It would be useful if the authors could include a feature that allows the TopAnat method to print the 41 genes which represent the paired limb/fin bud. At some point, the users might want to revisit their gene lists and tag their genes based on the different anatomical structures. Other tools that perform Enrichment tests, for example Enrichr, have this feature and this is extremely useful, in my opinion.*

 This is a good point. It is possible to cross the *geneList* vector with the expression mapping present in the *myTopAnatData* object. Another approach is to use functions that are inherited from the *topGO* package. For the “paired limb/fin bud” term:

 myTerm <- "UBERON:0004357"

 termStat(myTopAnatObject, myTerm)

 # 198 genes mapped to this term for Bgee 14.0 and Ensembl 84

 genesInTerm(myTopAnatObject, myTerm)

 # 48 significant genes mapped to this term for Bgee 14.0 and Ensembl 84

 annotated <- genesInTerm(myTopAnatObject, myTerm)[["UBERON:0004357"]]

 annotated[annotated %in% sigGenes(myTopAnatObject)]

 We have added this example at the end of the "Anatomical expression enrichment analysis" section.
